# Hyperspectral imaging to characterize the vegetative tissue biochemical changes in response to water deficit conditions in sorghum (*Sorghum bicolor*)

**DOI:** 10.3389/fpls.2025.1515998

**Published:** 2025-05-29

**Authors:** Yuvraj Chopra, Xinyan Xie, James Clothier, Souparno Ghosh, Hongfeng Yu, Harkamal Walia, Scott E. Sattler

**Affiliations:** ^1^ Department of Agronomy and Horticulture, University of Nebraska-Lincoln, Lincoln, NE, United States; ^2^ School of Computing, University of Nebraska-Lincoln, Lincoln, NE, United States; ^3^ Department of Statistics, University of Nebraska-Lincoln, Lincoln, NE, United States; ^4^ Wheat, Sorghum and Forage Research Unit, USDA-ARS, Lincoln, NE, United States

**Keywords:** hyperspectral imaging, water content, energy concentration, chemical sensing, machine learning, lignin, LASSO, water deficit

## Abstract

Hyperspectral imaging has been used to determine plant stress status. However, the biological interpretation of the spectral changes remain less explored. This can be addressed by building associations between stress-induced biochemical changes and variations in spectral reflectance. To this end, we tested spectral response of sorghum *brown midrib (bmr)* mutants under varying water stress levels using hyperspectral imaging (650–1650 nm). The *bmr* mutants have reduced lignin concentrations in their vegetative tissue which was reflected as spectral differences. Under water stress, the spectral signatures diverged more between the wildtype and mutants compared to control conditions. The genotype-dependent variation in spectral trends under water limitation was associated with differential sensitivity of the genotypes to water-limitation induced changes in energy density. We show that the energy density and relative water content of the plant tissue can be estimated accurately from spectral reflectance. To reduce the computational load, LASSO was used to obtain 22 wavelengths across the camera spectral range (650–1650 nm) in dried samples, to accurately predict energy density comparable to PLSR estimates. The reported wavelengths represent a useful screening tool for fast and reliable calorimetric estimations in bioenergy breeding programs.

## 1 Introduction

Major components of plant tissue, such as lignin, starch, lipids, carbohydrates, proteins, and water, have predominantly C-C, C-H, N-H, and/or O-H bonds. These bonds of organic molecules have different vibration response energies that constitute the aborption spectra (Sun, 2010). The interaction of incident radiation on a biological tissue is defined by the relative abundance of these compounds and their derivatives (Lu and Fei, 2014). The reflected transformations of the radiation generated because of interactions with different molecular structures are detected using spectroscopy with the aim of determining the biochemical composition of a plant tissue (Ferrari et al., 2004; Lu and Fei, 2014). Hyperspectral imaging (HSI) is one of the spectroscopic analysis platforms to capture and quantify reflected transformations over a continuous and wide range of the electromagnetic spectrum (Chakrabarti and Zickler, 2011). The hyperspectral image data constitutes a three dimensional hyperspectral cube (hypercube) that includes physical, geometric, and chemical/molecular information about the scanned material (Sun, 2010). This information is useful to characterize and identify plant biological macromolecules (Sytar et al., 2017). The spectral signature of a plant tissue is the combined signature of its complex biochemical composition (Sun, 2010). A specific wavelength cannot be uniquely associated with a particular compound because organic compounds absorb light at similar wavelengths (Curran, 1989), which presents a challenge for using traditional statistical regression methods for hyperspectral data due to high dimensionality and multicollinearity (Sun, 2010). Finding a correlation between multiple hyperspectral bands (predictor) and a single parameter of interest (predicted) requires detailed mathematical modeling (Singh et al., 2016). Machine learning models offer powerful tools to conduct complex mathematical modeling required to analyze hyperspectral data (Singh et al., 2016). Previously, these models have been used to correlate the biochemical components of plant tissues including water, macronutrients, micronutrients, cellulose, lignin, proteins, and secondary metabolites with hyperspectral signatures (Thulin et al., 2014; Pandey et al., 2017; Brugger et al., 2023).

Lignin is an essential component of plant tissue that provides structural integrity and helps conduct water through the vascular bundle. Lignocellulosic biomass, which is comprised of cell walls, is a renewable energy source for biofuels and renewable chemicals (Kyriakopoulos et al., 2010). However, lignin, an essential component of the plant cell wall, impedes conversion of cell wall polysaccharides to biofuels and decreases forage digestibility in ruminant livestock (Furtado et al., 2014). The *brown midrib* (*bmr*) mutants of sorghum and other C_4_ grasses are impaired in monolignol biosynthesis, the pathway that synthesizes hydroxycinnamyl alcohols, which are polymerized into lignin through radical coupled reactions within the cell wall (Vanholme et al., 2010). Sorghum *bmr* mutants, *bmr2-ref, bmr6-ref, bmr12-ref*, and “stacked” (*bmr6-ref bmr12-ref*), encode loss of function alleles of 4-coumarate:CoA ligase (4CL), cinnamyl alcohol dehydrogenase (CAD), caffeic O-methyl transferase (COMT), and both CAD and COMT, respectively. CAD and COMT are involved in the last two steps of monolignol biosynthesis (Bout and Vermerris, 2003; Sattler et al., 2009; Tetreault et al., 2018), whereas 4CL is located at the intersection of flavonoid, monolignol and hydroxycinnamic biosynthesis (Saballos et al., 2012). Lignin concentrations for the *bmr6-ref* and *12-ref* mutants were reduced in the vegetative tissues relative to wildtypes (Oliver et al., 2005b, a). The *bmr* mutants have reduced lignin concentrations (Porter et al., 1978; Oliver et al., 2005a, b; Sattler et al., 2010a). Mutant or population analysis for bioenergy purposes involves biochemical assessment of lignin and energy density, which are destructive and laborious. The lack of a rapid alternative screening tool to measure biochemical composition is a bottleneck in high-throughput genomics-assisted breeding strategies for sorghum (Pandey et al., 2017).

Disrupting lignin biosynthesis can affect the plant response to water limitation (Saluja et al., 2021). This observation can be used to explore the effects of inherent biochemical differences as well as the effects of water limitation on spectral response using HSI. Numerous studies have used HSI as a tool to characterize healthy and stressed plant tissue. However, the biological relevance of the observed spectral differences remain less explored. Spectral differences between healthy and stressed plant tissues correspond to stress-induced biochemical changes (Lu and Fei, 2014). Understanding of the relationship between stress-induced biochemical differences and their associated changes in spectral signatures is limited. Water deficit treatment of *bmr* mutants could generate a larger diversity among the biochemical composition of vegetative tissues and strengthen training sets for prediction models. The compositional diversity among the biomass of *bmr* mutants could be used to generate prediction models for developing HSI-based tools for rapid alternative biochemical assessment of sorghum vegetative tissue. Plant responses to water limitation include but are not limited to changes in water content, lignin, cellulose, hemicellulose, starch, and soluble sugars (Emerson et al., 2014; Perrier et al., 2017). Energy concentration or gross energy density is an analytical assay to gauge cumulative change in the biochemical composition of the plant tissue (Gardner et al., 2015). Lignin has a greater energy density than the other cell wall components (Novaes et al., 2010), and an increase in lignin or its related cell wall moieties is reflected in increased energy value (concentration) with the biomass (Gardner et al., 2015; Tetreault et al., 2018). Therefore, gross energy density can be an effective proxy for lignin content as well as detect biochemical changes induced by water limitation.

In this study, we sought to establish a correlation between stress-induced biochemical changes and their corresponding spectral responses. For this the absorption spectra from major organic compounds was cataloged and compared against reported NIRS (near-infrared spectroscopy) based absorption wavelengths. Next we focused on the effect of plant tissue dehydration on spectral reflectance. This information was then used to detect the altered spectral response of sorghum *bmr* mutants under water limitation, which could be attributed to differential sensitivity to water limitation-induced biochemical changes. Finally, predictive wavelengths were identified for energy density by using the prediction models. To reduce computational burden for energy density estimations, LASSO was used to obtain a subset of predictive wavelengths. The models trained for this analysis represent a rapid and accurate approach for gross energy density estimation in plants. Overall, this study advances our understanding of the HSI for stress and chemical sensing in plants.

## 2 Materials and methods

### 2.1 Plant materials

Sorghum *bmr* mutants, *bmr12-ref* (COMT), *bmr6-ref* (CAD), *bmr2-ref* (4CL), and “stacked” (*bmr6-ref bmr12-ref*) near-isogenic lines in the RTx430 background were used for the study (Pedersen et al., 2006; Saballos et al., 2012). RTx430 was used as the wild type for this study. Hereafter, *bmr12-ref*, *bmr6-ref*, *bmr2-ref*, (*bmr6-ref bmr12-ref*) and wildtype are referred to as *bmr12*, *bmr6*, *bmr2*, “stacked”, and WT, respectively.

### 2.2 Plant growth conditions and treatments

Seedlings were germinated and grown using the cigar roll method, as described earlier (Zhu et al., 2006; Saluja et al., 2021). Surface sterilized seeds (1 min in 70% ethanol, 5 min in 50% bleach containing 0.1% Triton X-100 (Sigma-Aldrich, St. Louis, MO), 1 min in 70% ethanol, and 6–7 rinses with sterile deionized water), were placed on moist germination paper in Petri plates incubated at 25°C in the dark for 2 days. Uniform seedlings were selected from the germinated set, and five seedlings were placed in each cigar roll. 30 seedlings per genotype in cigar rolls were placed in a 1 Liter beaker. 200ml of one-tenth strength Hoagland solution (Hoagland and Arnon, 1950), was used as nutrient medium for the cigar roll assays. Seedlings were grown at 28°C/25°C, 13hr/11hr, day/night at 40-50% relative humidity in a controlled system incubator for 6 days. At 6 days, seedlings were transplanted to the greenhouse on the East Campus of the University of Nebraska – Lincoln (UNL) (lat. 40°50’N, long. 96°39’W). Plants were grown in Classic 1000c (Nursery Supplies) = 25.71 x 23.17 x 20.63 cm pots; a total of 87 pots were filled with the same amount (6.8 kg) of standard greenhouse mix from the same batch. The standard greenhouse mix was comprised of 38.5% peat, 23.1% soil, 19.2% sand, and 19.2% vermiculite. The greenhouse conditions were set at temperature 30/27 ± 1°C, and light/dark 12/12h. For the first 21 days, all pots were maintained at 80-90% water holding capacity (WHC) using fertigation. Fertigation applications were made using Peters^®^ Professional General Purpose (20-10-20) fertilizer at 250ppm. After 21 days, water was withheld until the desired treatment level (WHC) was achieved. For calculating the WHC of the soil mix, the difference between oven-dried and soil at field capacity was recorded. For oven-dried weights, three soil mix samples of 6.8 kilograms each were oven-dried (60°C for 14 days). The soil was then transferred to pots with drainage holes at the bottom. The soil was saturated with water and covered at the top to prevent evaporative losses. The pot weights were then recorded at regular intervals until no change in pot weight was observed and there was no free-flowing water from the drainage holes. To test the spectral response of sorghum to a range of water availability, four treatment levels were defined as 80% WHC (well-watered), 60% WHC, 40% WHC, and 30% WHC (water-deficit). Once the desired WHC capacity was achieved, additional water was supplied every other day to maintain treatment levels based on pot weight.

### 2.3 Experimental design

The experimental design included four treatment levels, five genotypes, and four replicates per genotype per treatment (**Supplementary Figures S1**, **S2**). To ensure that the design allowed for the control of genetic variability and that each genotype was well represented within each treatment group, the overall experimental design for the layout was Randomized Complete Block Design. We assigned a single treatment to each block, and within each block, a complete randomized design was adopted for the five genotypes. Four rectangular blocks, each containing 21 pots (7×3), were laid out next to each other. Each block also contained one treatment control pot (no plant). The blocks were maintained at defined treatment levels until 90 days after transplanting (DAT).

### 2.4 Sampling of plant tissue for hyperspectral imaging

**Figure 1:**
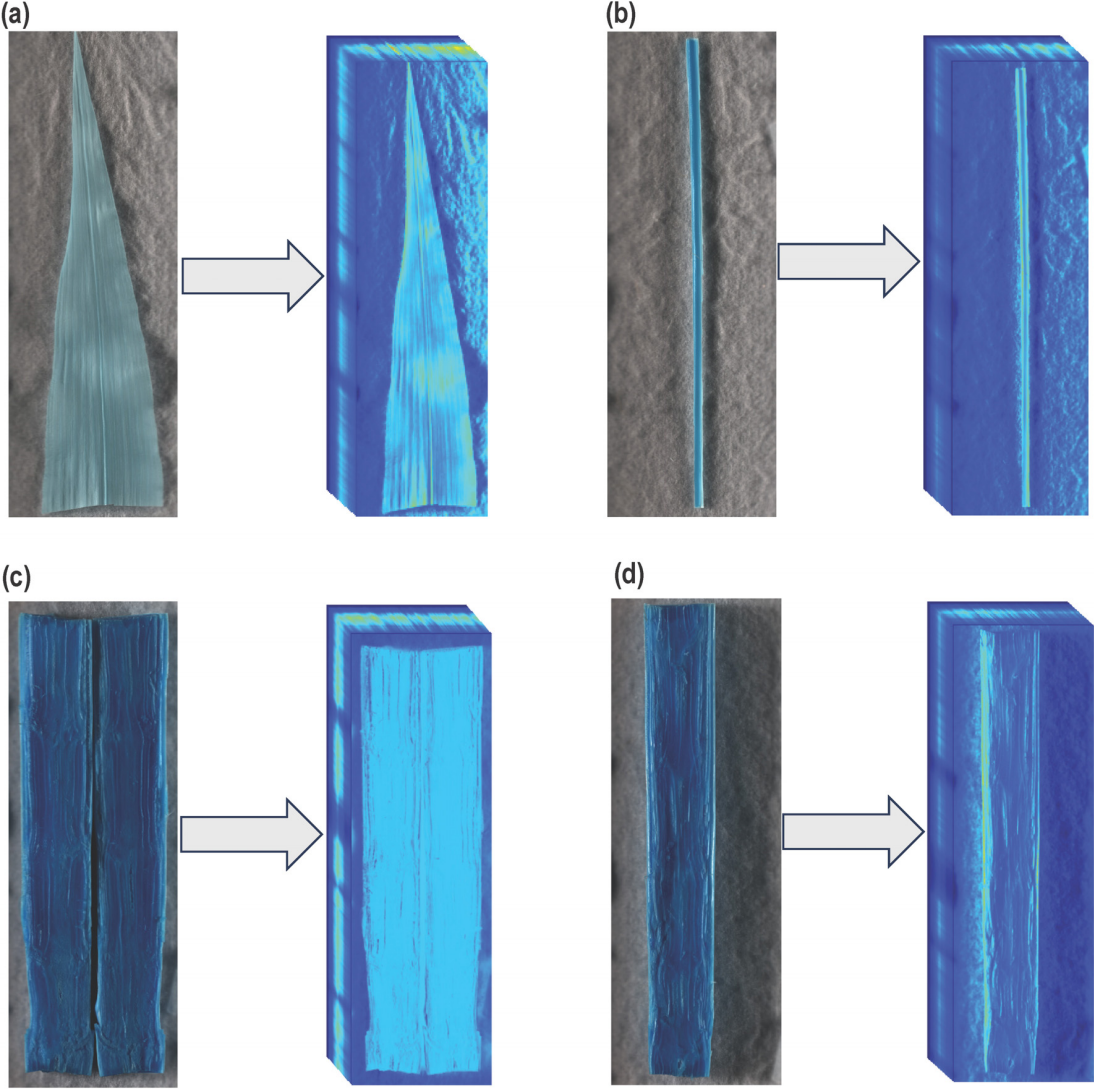
Generated Hypercube of tested samples: **(a)** Abaxial surface of leaf **(b)** Adaxial surface of midrib **(c)** Full section of stalk tissue **(d)** Half section of stalk tissue.

During this experiment, three different types of plant tissue, including leaf samples, midrib samples, and stalk samples, were imaged for their spectral response. A section 15 cm from the leaf tip was harvested from the youngest fully expanded leaf for a leaf sample. Mature midrib samples were collected as a 15 cm section from the base of the leaf towards the tip with leaf lamina removed. Stalk samples comprised a 15 cm section harvested above the crown. Four Hyperspectral Imaging (HSI) time points, HSI-1, HSI-2, HSI-3, and HSI-4, corresponded to 38, 60, 83, and 89 DAT. At HSI-1, all treatments reached the desired treatment level except 30% WHC, which needed to further dry down. Therefore, all treatments except 30% WHC were imaged at this time point. Leaf samples were collected at HSI-1, HSI-2, and HSI-3. At HSI-3, due to prolonged water limitation and stunted growth under 40% and 30% WHC, the second youngest fully expanded leaf was selected for sampling. Midrib samples were collected only at HSI-3. At the final imaging session (HSI-4), only stalks were sampled. The sample sizes and methods were consistent for all the samples across all the blocks. **Figure 1** illustrates the 2D images and the corresponding hypercubes of example plant parts. The 2D images allow for visual examination of the data, supporting informed decision-making, while the hypercubes capture and quantify the spatial and spectral characteristics of the plant tissues across multiple wavelengths.

### 2.5 Hyperspectral imaging of vegetative tissue

Both the adaxial and abaxial surfaces of a leaf (**Figure 1a**) or midrib (**Figure 1b**) sample were scanned, and the average value of spectral reflectance was used for further analyses. The stalk samples were carefully split longitudinally into two equal sections using a scalpel. The pieces of the split stalk were placed next to each other on the flatbed platform, with sections facing the camera (**Figure 1c**). The stalk sample results generated from the aforementioned technique were denoted as “Full section results”. As a technical replicate and to rule out the possibility of interference in Full section results due to shadowing between the two pieces of a sample, the split sections were also tested individually. For a given sample, each half of the stalk was imaged and processed separately, and the spectral reflectance for both halves were averaged for each sample. The stalk sample results generated from this method were described as “Half section results” (**Figure 1d**). All the results from this study should be considered “Full section results” unless otherwise mentioned. After collection, the samples were immediately subjected to imaging (0 hours = fresh imaging), and the fresh weights were also recorded. For leaf and midrib samples, following the fresh imaging, the samples were oven-dried at 55°C for 14 days, after which they were imaged again, and the dried weights were recorded. For stalk samples, a uniform drying technique was used to understand the effect of dehydration on spectral response at different drying stages. After fresh imaging, the split stalk samples were labeled and placed on a 38.1 × 33.02 cm plastic tray inside a bench-mounted fume hood (Lab Crafters Inc.) at room temperature for 120 hours. The fume hood at room temperature had a continuous airflow at face velocity of approximately 0.59 m/s. During the drying process, samples were imaged and weighed at regular intervals starting 6, 12, 24, 48, 72, 96, 103, and 120 hours after harvesting (HAH). The ambient drying allowed approximately 90 - 95% of the total water content to evaporate (percentage evaporation); sample weights stopped changing at 120 HAH. Percentage evaporation (PE) was calculated as,


$$\text{PE}=\frac{FW-OW}{FW-DW}\;\times 100$$

where OW is the weight of the sample at 120 HAH, DW is the dry weight of the sample measured after oven drying the sample, and FW is the fresh weight of the sample measured right after harvest. After 120 HAH to remove bonded water molecules, samples were oven-dried at 55°C for 7 days, following which they were imaged and weighed again. Sample weights were recorded before each imaging session to calculate the sample water content as a percentage of dry weight (SWC). SWC was then derived using the equation,


$$\text{SWC}=\frac{FW-DW}{DW}\;\times 100$$

A near perfect correlation was reported for SWC and Relative water content of the sample (RWC) (Kovar et al., 2019). Relative water content (RWC) was calculated as,


$$\text{RWC}=\;\frac{FW-DW}{SW-DW}\;\times 100\;$$

where SW is the saturated weight of the sample measured after overnight water saturation. In our study, the terms RWC and SWC are used interchangeably, both defined by the same equation,


$$\text{RWC or SWC}=\frac{FW-DW}{DW}\;\times 100$$

### 2.6 Bomb calorimetry

The stalk samples after oven-dried imaging were ground in a SPEX SamplePrep Geno/Grinder^®^ 2010. The ground samples were subjected to calorimetric measurements to determine energy concentrations using a Parr 6400 bomb calorimeter (Parr Instrument Co.). As described (Tetreault et al., 2021), approximately 200 milligrams (mg) of dried ground stalk tissue combined with 600 mg of mineral oil were combusted to estimate calories per gram (cal/g) of dry weight (Calories gram^-1^). Energy values were calculated by subtracting the energy released from the combustion of only the mineral oil, from the combined combustion of mineral oil and stalk, which was standardized to the sample weight.

### 2.7 Organic compound standards

Cotton fiber (at least 90% cellulose) was imaged as a cellulose standard (Felgueiras et al., 2021). Corn starch (ARGO^®^ corn starch) was imaged as a starch standard. Lignin extracted from sorghum forage using filter bag method was imaged as purified acid detergent lignin from sorghum (Vogel et al., 1999). Casein acid hydrolysate (Sigma^®^ C-9386) and casein enzymatic hydrolysate (Sigma^®^ C-7290) were used as the protein standards. Urea (Mallinckrodt GenAR^®^ 7729) was imaged as a fixed nitrogen standard.

### 2.8 Imaging platform and image processing

The imaging platform used in this experiment is designed and engineered as described (Gao et al., 2021). The imaging platform used a high-performance line-scan image spectrograph (Micro-Hyperspec^®^ Imaging Sensors, Extended VNIR version, Headwall Photonics, Fitchburg, MA, USA) with spectral range extending 600–1700 nm at 5.5 nm spectral resolution. To obtain the precise hyperspectral curve of the sample, a comprehensive multi-step process was used in MATLAB, including derivation of an initial sample mask through segmentation of the plant tissue from the original image, refinement of the initial mask, applying the sample mask to the entire hyperspectral matrix, calibration of the original hyperspectral intensity $\left(I_{O}\right)$ , and averaging the hyperspectral value across the tissue’s area.

The CIELAB color space was utilized to derive the initial mask, commonly denoted as L*a*b*. Here, ‘L*’ signifies the lightness, with values extending from 0 (representing black) to 100 (indicating white). The ‘a*’ axis delineates the chromaticity between green and red, where values range from -100 (green) to 100 (red). Similarly, the ‘b*’ axis demarcates the chromaticity between blue and yellow, extending from -100 (blue) to 100 (yellow). This color space’s intrinsic characteristics facilitate the differentiation between the plant object and the background, offering a more effective segmentation than that achieved using the RGB color space. Value ranges for each axis were manually chosen to minimize background and maximize the sample coverage, which enabled the generation of a binary image that incorporated plant tissue segments with non-uniform color distribution. The mask was refined using a disk-shaped structuring element to fill connected plant tissue regions, and morphological operations were employed to address any holes, with the operation’s scope defined by the neighborhood’s shape and size around each pixel. The refined mask was applied to each channel of the loaded hyperspectral matrix, which encapsulates hyperspectral intensity across 242 channels for each pixel. Calibration of the original hyperspectral intensity $\left(I_{O}\right)$ was used to correct systematic discrepancies in the imaging process, variances in sensor performance across different devices, and the necessity of wavelength calibration. This correction ensured sensor accuracy and facilitated the comparisons of hyperspectral data across sensors and over time. Calibration involved obtaining a white reference $\left(I_{w}\right)$ using a spectrally flat target under consistent lighting and a dark reference $\left(I_{d}\right)$ by capturing an image with the camera’s shutter closed. The calibrated hyperspectral intensity $\left(I_{c}\right)$ was then derived using the following equation, which integrates these references to adjust the hyperspectral data accurately.


$$I_{c}=\frac{I_{o}-I_{d}}{I_{w}-I_{d}}$$

The final intensity value for each channel was calculated by aggregating the intensity values of all sample pixels within a channel and then dividing them by the total number of pixels to derive the average. This process was repeated for each spectral channel, thereby yielding a hyperspectral curve across the entire spectrum.

### 2.9 Image acquisition

To acquire images, the camera controlling software HyperSpec^®^ III (Headwall Photonics, Fitchburg, MA, USA) was calibrated (Gao et al., 2021) at an Exposure level of 12ms, a Frame period of 18ms, and the Gain level set at low. After calibration, the Exposure was changed to 6ms, while other settings remained unchanged. The samples were scanned in Free Run mode with Images per frame set at 1, and frames per cube set to 2010. The source FPS and write FPS were 55.55. The platform speed was set to 1% under the manual mode of video shot settings on the controller. A total of 3,773 images were recorded with a combined size of 5.8 terabytes.

### 2.10 Packages for statistical analysis

Ribbon plots and box plots were generated using the R packages ‘ggplot2’ (Wickham, 2011) and ‘reshape2’ (Wickham, 2007). Prediction modeling, Clustering, and Classification were also performed using R packages. R packages ‘pls’ (Mevik and Wehrens, 2007), ‘e1071’ (Dimitriadou et al., 2009), ‘randomForest’ (Breiman et al., 2021) were used for partial least squares regression (PLSR), support vector machine (SVM) and random forest (RF) respectively. The ‘glmnet’ (Friedman et al., 2010) R package was used for LASSO regression, and the ‘cluster’ (Maechler, 2018) R package was used for clustering.

### 2.11 Clustering and classification

Exploratory K-means clustering was performed on the training data to determine treatment structure in relation to PLSR components. The treatment identifiers were not included as part of the predictive mechanism in the training of the PLSR model.The optimal number of clusters *k=4* was selected using the gap-statistic. The gap-statistic reference distribution was created using 500 bootstrap samples. Clusters were initialized with 25 random starting centers to ensure optimal centering of the final clusters. The components naturally clustered by treatment, and observations included in each of the clusters belonged to the same treatment group. For the classification of treatments using PLSR scores, SVM C-classification with the radial basis function as the kernel was performed. Hyperparameters were selected using a coarse grid search. The values for gamma and the cost were selected to minimize the cross-validation classification error.

### 2.12 Machine learning based predictions

The data contained 242 predictors, representing the 242 wavelengths from hyperspectral imaging. For model selection and estimation of prediction accuracy, the energy concentration data were split into a training and a test set consisting of 60 and 16 observations (75/25 split), respectively. The training set was further split into 5 folds for hyperparameter selection. Candidate predictive models were PLSR, RF, LASSO, and SVM epsilon regression using the radial basis function as the kernel. Hyperparameters were chosen to minimize the cross validation root mean squared error (RMSE_CV_). The hyperparameters tuned consisted of the number of components for the PLSR, the number of variables sampled at each split and the size of the leaf for the RF, the lambda (penalty strength) for LASSO, and the gamma value and cost for the SVM. For RF and SVM, a coarse grid search was used to select the optimal hyperparameters. The final models were fitted to the training data, and the out of sample prediction error was estimated using the test set.

The aforementioned analysis was also applied for the RWC prediction, with the RWC data split into a training and a test set consisting of 530 and 132 observations (80/20 split), respectively. For each treatment level prediction models, the RWC data was split into a training and a test set consisting of 124 and 42 observations (75/25 split), within each treatment. The oven dried samples had zero RWC, therefore excluded from RWC predictions. The prediction equations generated in this study use spectral data of only stalk samples.

## 3 Results

### 3.1 Spectral signatures of standards of major organic compounds

**Figure 2:**
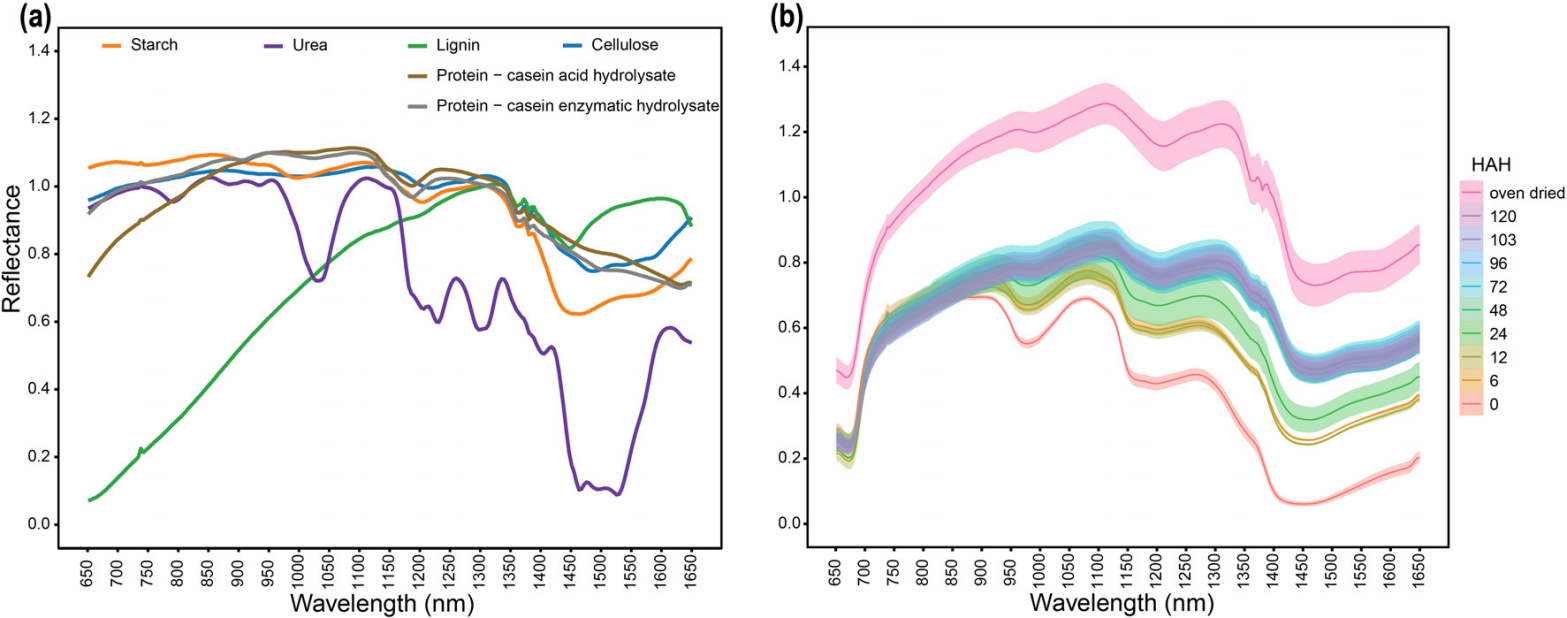
Spectral signatures of major organic compounds in the spectral range 650–1650 nm **(a)** Spectral signatures of pure standard of major organic compounds **(b)** Uniform drying-based spectral response of *bmr12* stalk samples from 80% WHC showing the relationship between tissue hydration and spectral response; HAH indicates hours after harvesting. The solid line represents the mean of the samples, and the ribbon around represents Standard Error (SE).

To examine the ability of the HSI system to differentiate macromolecules and understand their spectral trends in isolation, we first imaged and analyzed standards for the major organic compounds. The hyperspectral signature of the tested compounds showed unique spectral patterns (**Figure 2a**). To test the detection of C-H, O-H bond vibrations, purified carbohydrate samples were used. Carbohydrate samples, including starch and cellulose, had similar signatures; however, there were detectable differences between starch and cellulose in the 1400–1650 nm range. Starch absorption features were observed around 950–1000 nm, 1200 nm, and 1400–1650 nm. Conversely, absorption features for cellulose were observed around 1200 nm and 1400–1650 nm. Plants mainly store nitrogen in the form of nitrate and proteins. To determine the spectral signature of proteins, two animal protein standards were tested. Casein acid hydrolysate and casein enzymatic hydrolysate had similar spectral properties including absorption features around 1100–1200 nm and 1400–1650 nm. A similar absorption range for the tested protein standards, which was distinguishable from the absorption range of carbohydrates, indicated that our setup was able to differentiate between distinct classes of macromolecules along with reporting similarities between different derivates of the same protein sample.

Lignin was used as a standard for C-C bond vibrations. Lignin had a distinct spectral signature characterized by exceptionally low reflectance in the 650–1100 nm range and a strong reflectance feature in the 1450–1600 nm range (**Figure 2a**). Conversely, absorption features for lignin were observed at 1450 nm and around 1650 nm (**Figure 2a**). The observed wavelengths have been associated with lignin absorption using NIRS previously (Curran, 1989). To further validate the system for C-N and N-H bond vibrations, urea samples were tested. Glutamine and asparagine are important nitrogen transporters and storage compounds due to their high nitrogen to carbon ratio (2:4) (Lea et al., 2007); urea shares structural similarity with asparagine and has a higher nitrogen to carbon ratio (2:1) (Vaughan and Donohue, 1952). Urea provides improved detection of N-H bond vibrations with minimal noise from C-N or C-O vibrations when compared with asparagine. Therefore, urea was used as nitrogen standard. Urea had a unique spectral pattern with multiple absorption features across the spectral range (**Figure 2a**); a significantly strong absorption feature was detected between 1450–1525 nm (**Figure 2a**). Wavelenght corresponding to 1510 nm has been reported for nitrogen absorption feature using NIRS (Curran, 1989). Overall, the wavelength range 650–1100 nm and beyond 1500 nm represents regions where all tested samples had some level of separation (**Figure 2a**); however, in the 1100–1500 nm range most organic compounds had a similar spectral response. Within this region, a spectral range between 1350–1400 nm was particularly noteworthy because all tested samples (except urea) shared the same absorption and reflectance features (**Figure 2a**). Therefore, the 1350–1400 nm spectral range had high levels of ambiguity because the spectral signatures of different biochemical components were overlapping at this range. Collectively, these results show the spectral regions with overlapping and non-overlapping absorption features among different organic compounds.

### 3.2 Spectral changes in response to tissue hydrations

To examine the spectral signature of a plant tissue with varying levels of dehydrations, we used a dry down approach. The drying process shifted reflectance curves upwards, exhibiting increments in mean reflectance of the stalk samples due to dehydration (**Figure 2b**). The response of stalk samples to drying was similar for all the genotypes; drying curves for other genotypes from 80% WHC (well-watered treatments) are shown in **Supplementary Figure S3**. In oven-dried samples, the 1350–1400 nm absorption feature matched the spectral signature for organic compounds (**Figure 2a**), which was otherwise masked in fresh samples (0 HAH). The response of reflectance curve to dehydration followed a simple linear relation, where for most of the spectrum, the reflectance intensity was directly proportional to level of dehydration. The absorption features related to the biochemical composition of a sample became more evident as the water evaporated. Therefore, oven drying eliminated interference in the spectral signatures otherwise created by water, and oven-dried samples are suitable for spectral-based compositional determination.

### 3.3 Changes in spectral properties of the vegetative tissue in response to water limitation

**Figure 3:**
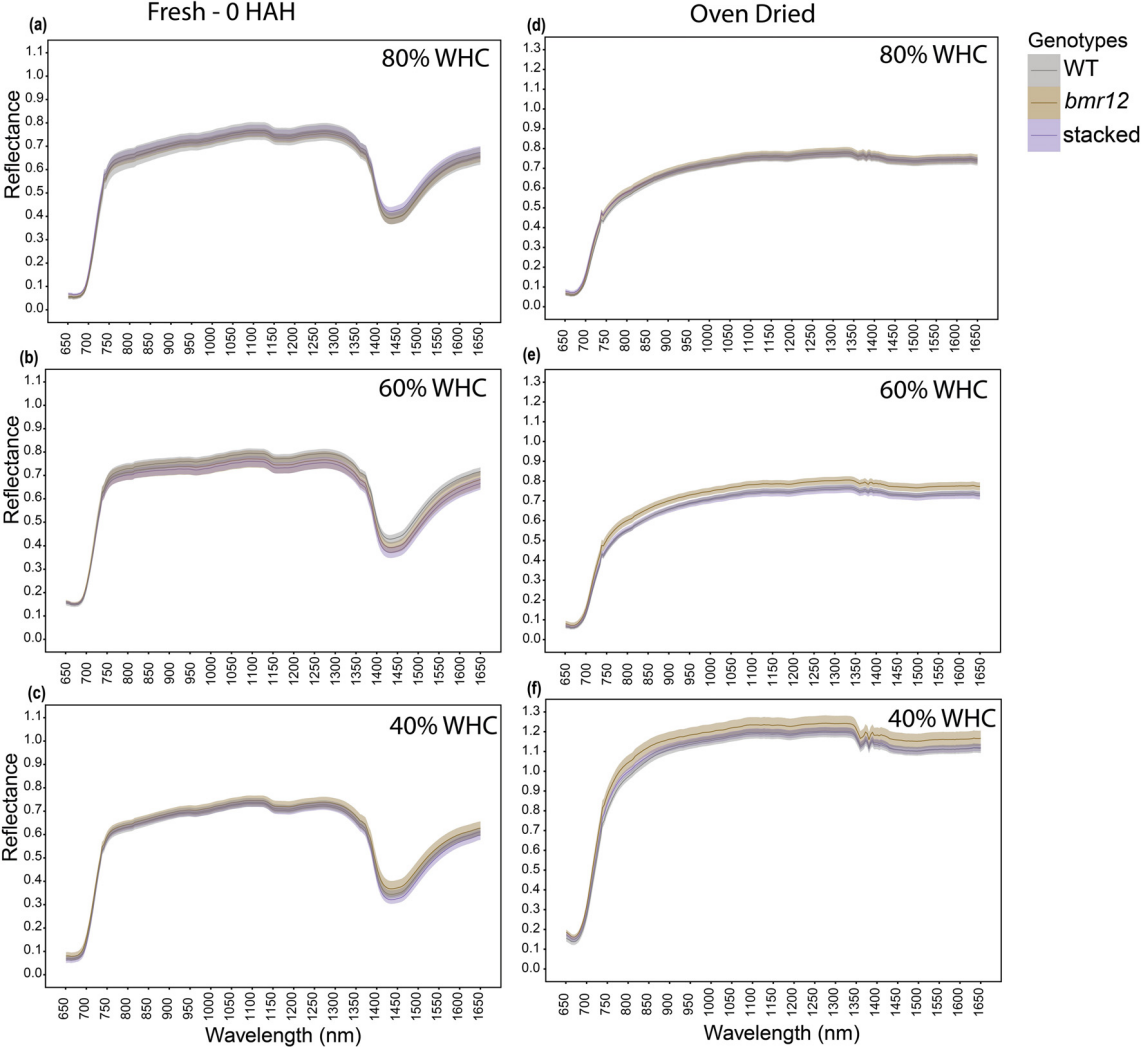
The averaged leaf spectral signatures of genotypes at HSI-1 (38 DAT) under different water treatments and imaging conditions in the spectral range 650–1650 nm (n = at least 3 for each group): **(a)** Spectral reflectance of genotypes at 80% WHC for fresh imaged samples **(b)** Spectral reflectance of genotypes at 60% WHC for fresh imaged samples **(c)** Spectral reflectance of genotypes at 40% WHC for fresh imaged samples **(d)** Spectral reflectance of genotypes at 80% WHC for samples imaged after oven drying **(e)** Spectral reflectance of genotypes at 60% WHC for samples imaged after oven drying **(f)** Spectral reflectance of genotypes at 40% WHC for samples imaged after oven drying; HAH and WHC indicate hours after harvesting and water holding capacity, respectively. The solid line represents the mean of the samples and the ribbon around represents Standard Error (SE).

To examine how biochemical differences in vegetative tissues affect spectral signatures in response to water limitation, *bmr* mutants were grown under four water treatment levels. To simplify visual comparisons, we focused only on three genotypes selected as highest (WT), lowest (stacked), and an intermediate value (*bmr12*) of lignin content at maturity (Sattler et al., 2010b). Leaf blades were imaged at HSI-1 (38 DAT). For fresh imaged samples (**Figures 3a–c**), the spectral signatures of the genotypes overlapped across the spectrum under all treatments with one exception observed across the treatments between 1350–1650 nm. The spectral reflectance trends among the genotypes were unique under each treatment. The differences in mean reflectance for the genotypes were larger as compared to the SE (standard error) of the means for the genotypes. For samples imaged after oven drying (**Figures 3d–f**), 80% WHC had no significant differences among the spectral signatures of the genotypes (**Figure 3d**). At 60% and 40% WHC (**Figure 3e**), the spectral signature for *bmr12* had a clearly observable separation from WT and stacked. Although the separation among spectral signatures was limited for leaf samples at HSI-1, there were features suggesting unique spectral responses of the genotypes. As observed for oven-dried samples, across the treatments *bmr12* had the highest reflectance followed by WT and stacked; the spectral signatures of WT and stacked overlapped across the spectrum. Conversely, the spectral trends among the genotypes were treatment dependent for fresh imaged samples.

**Figure 4:**
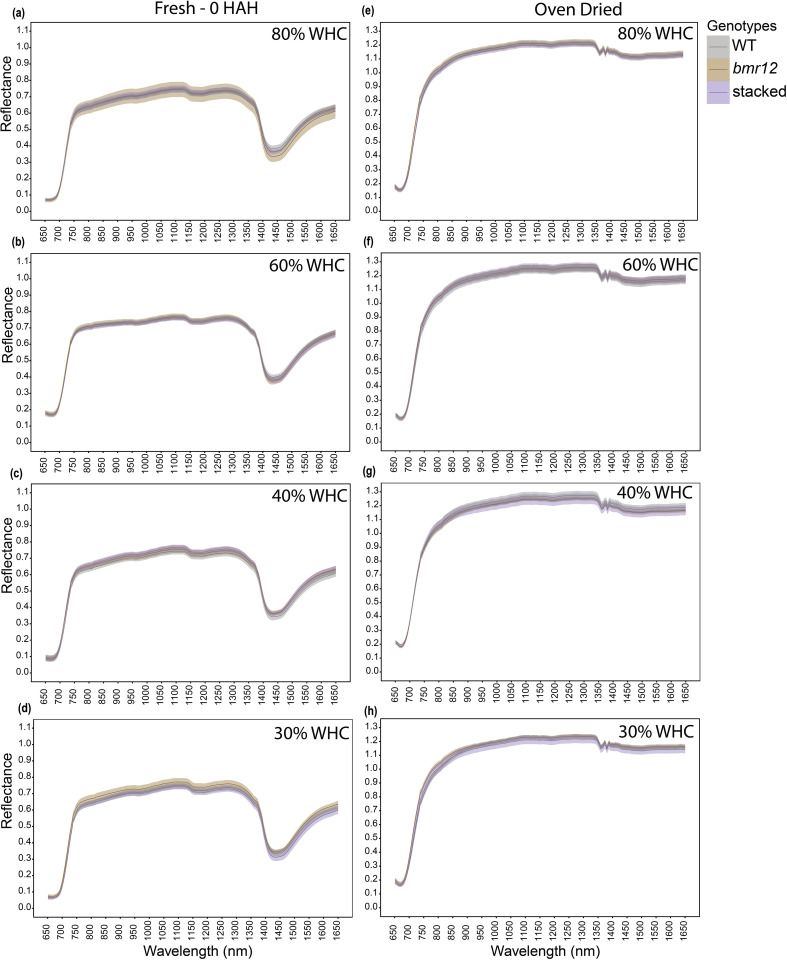
The averaged leaf spectral signatures of genotypes at HSI-2 (60 DAT) under different water treatments and imaging conditions in the spectral range 650–1650 nm (n = at least 3 for each group): **(a)** Spectral reflectance of genotypes at 80% WHC for fresh imaged samples **(b)** Spectral reflectance of genotypes at 60% WHC for fresh imaged samples **(c)** Spectral reflectance of genotypes at 40% WHC for fresh imaged samples **(d)** Spectral reflectance of genotypes at 30% WHC for fresh imaged samples **(e)** Spectral reflectance of genotypes at 80% WHC for samples imaged after oven drying **(f)** Spectral reflectance of genotypes at 60% WHC for samples imaged after oven drying **(g)** Spectral reflectance of genotypes at 40% WHC for samples imaged after oven drying **(h)** Spectral reflectance of genotypes at 30% WHC for samples imaged after oven drying; HAH and WHC indicate hours after harvesting and water holding capacity, respectively. The solid line represents the mean of the samples and the ribbon around represents Standard Error (SE).

For HSI-2 of leaf blades at 60 DAT, spectral signatures of the fresh imaged samples (**Figures 4a–d**) overlapped across the spectrum for all genotypes under all treatments; some separation was observed between 1350–1650 nm. The spectral trends for 80% and 60% WHC were similar, whereas trend shifts were observed under water deficit conditions. At 30% WHC (**Figure 4d**), mean reflectance among the samples was more distinct compared to other treatments. For samples imaged after oven drying (**Figures 4e–h**), the spectral signatures of the genotypes overlapped under all treatment levels. The observations at HSI-2 were similar to HSI-1 in terms of overlapped signatures across the spectrum and separation observed between 1350–1650 nm across treatments. Unlike HSI-1, spectral trends were not conserved at HSI-2 for oven-dried samples.

**Figure 5:**
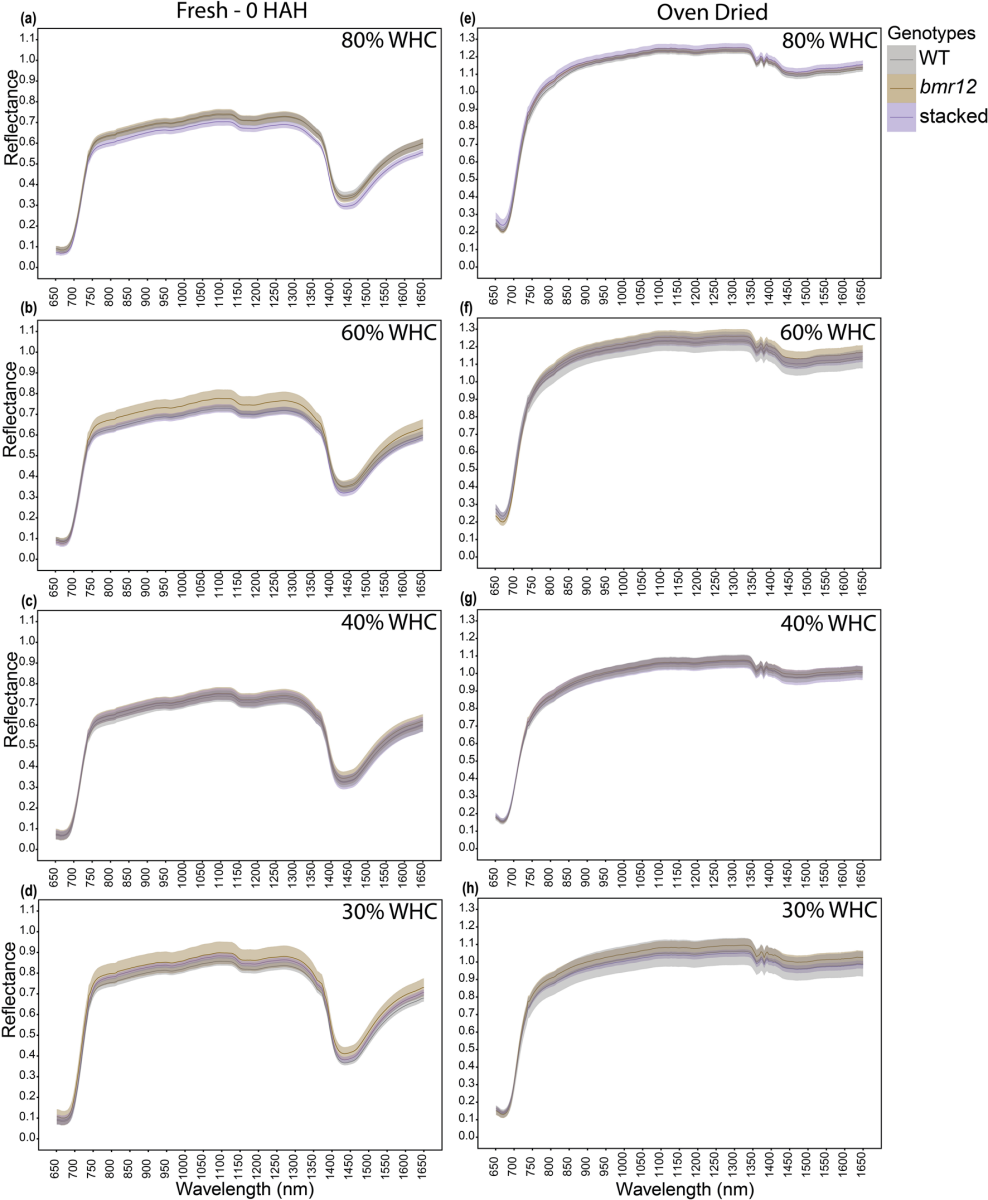
The averaged leaf spectral signatures of genotypes at HSI-3 (83 DAT) under different water treatments and imaging conditions in the spectral range 650 – 1650nm (n = at least 3 for each group): **(a)** Spectral reflectance of genotypes at 80% WHC for fresh imaged samples **(b)** Spectral reflectance of genotypes at 60% WHC for fresh imaged samples **(c)** Spectral reflectance of genotypes at 40% WHC for fresh imaged samples **(d)** Spectral reflectance of genotypes at 30% WHC for fresh imaged samples **(e)** Spectral reflectance of genotypes at 80% WHC for samples imaged after oven drying **(f)** Spectral reflectance of genotypes at 60% WHC for samples imaged after oven drying **(g)** Spectral reflectance of genotypes at 40% WHC for samples imaged after oven drying **(h)** Spectral reflectance of genotypes at 30% WHC for samples imaged after oven drying; HAH and WHC indicate hours after harvesting and water holding capacity, respectively. The solid line represents the mean of the samples and the ribbon around represents Standard Error (SE).

The final imaging timepoint (HSI-3) for leaf blades was at 83 DAT. For fresh samples, at 80% WHC (**Figure 5a**), stacked mutant had a distinct and non-overlapping spectral signature. At 60% WHC (**Figure 5b**), *bmr12* signature separated from other genotypes. At 40% WHC (**Figure 5c**), spectral signatures for the genotypes were almost indistinguishable. At 30% WHC (**Figure 5d**), mean reflectance for the genotypes was more distinct than that of other treatment levels. For samples imaged after oven drying (**Figures 5e–h**), similar to observations at HSI-1 (**Figure 3**) and HSI-2 (**Figure 4**), the spectral signatures of the genotypes completely overlapped across the spectrum under all treatment levels except 30% WHC (**Figure 5h**), where mean reflectance for the genotypes was more distinct compared to other treatment levels. For leaf blade imaging, the spectral trends across growth stages were treatment dependent. At HSI-3, the differences among the leaf blade spectral signatures of genotypes were more significant when compared to HSI-1 and HSI-2. This indicated that the spectral signatures of genotypes exhibited greater differences when leaf blades were imaged at the end of the vegetative stage; therefore, imaging leaf blades at this stage is more suitable for detecting spectral differences among the genotypes.

**Figure 6:**
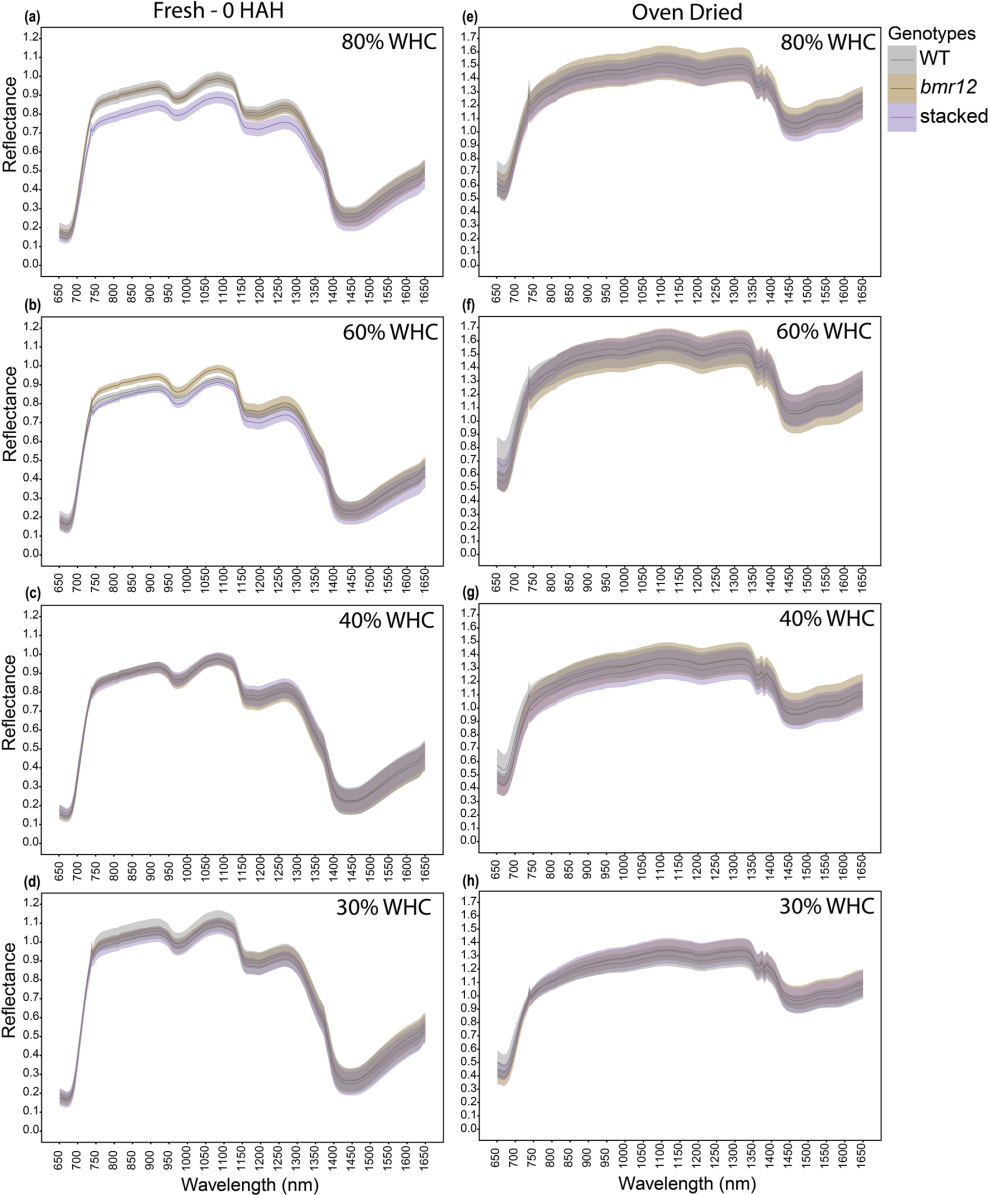
The averaged midrib spectral signatures of genotypes at HSI-3 (83 DAT) under different water treatments and imaging conditions in the spectral range 650–1650 nm (n = at least 3 for each group): **(a)** Spectral reflectance of genotypes at 80% WHC for fresh imaged samples **(b)** Spectral reflectance of genotypes at 60% WHC for fresh imaged samples **(c)** Spectral reflectance of genotypes at 40% WHC for fresh imaged samples **(d)** Spectral reflectance of genotypes at 30% WHC for fresh imaged samples **(e)** Spectral reflectance of genotypes at 80% WHC for samples imaged after oven drying **(f)** Spectral reflectance of genotypes at 60% WHC for samples imaged after oven drying **(g)** Spectral reflectance of genotypes at 40% WHC for samples imaged after oven drying **(h)** Spectral reflectance of genotypes at 30% WHC for samples imaged after oven drying; HAH and WHC indicate hours after harvesting and water holding capacity, respectively. The solid line represents the mean of the samples and the ribbon around represents Standard Error (SE).

At HSI-3, differences among the leaf midrib-based spectral signatures of genotypes were more obvious for fresh samples (**Figures 6a–d**). At 80% WHC (**Figure 6a**), stacked had a distinct and non-overlapping spectral signature. At 60% WHC (**Figure 6b**), all genotypes had clear separation among their spectral signatures, where *bmr12* had the highest reflectance, followed by WT, and then stacked for most of the spectrum. At 40% (**Figure 6c**) and 30% WHC (**Figure 6d**), spectral signatures for all the genotypes overlapped. For oven-dried samples (**Figures 6e–h**), differences among mean reflectance of the genotypes were higher than the SE. At 80% WHC (**Figure 6e**), the spectral trends were comparable to fresh imaging results (**Figure 6a**). At 60% WHC (**Figure 6f**), the spectral trends were reversed compared to 80% WHC (**Figure 6e**), where stacked had the highest mean reflectance followed by *bmr12*, and WT overlapped for most part of the spectrum. At 40% WHC (**Figure 6g**), spectral trends matched observations at 80% WHC (**Figure 6e**). The resolution of differences among the genotype means was higher for oven-dried samples at 40% WHC compared to its fresh imaging counterpart. At 30% WHC (**Figure 6h**), a unique trend was observed where spectral signatures of *bmr12* and stacked overlapped across the spectrum and had higher reflectance than WT. Within the category of foliar tissue (leaf blade and isolated midrib), spectral differences among the genotypes had better resolution when midrib samples were used for imaging. The spectral signatures of the genotypes across growth stages had better separation when samples were imaged fresh; conversely, midrib samples from water deficit treatments had better separation when imaged after oven drying. The leaf midrib-based spectral trends from fresh imaging at 80% WHC and 60% WHC (**Figures 6a, b**) matched leaf blade-based fresh imaging spectral trends for the same treatments (**Figures 5a, b**). This result emphasizes the consistency of the system in detecting differences among the genotypes.

**Figure 7:**
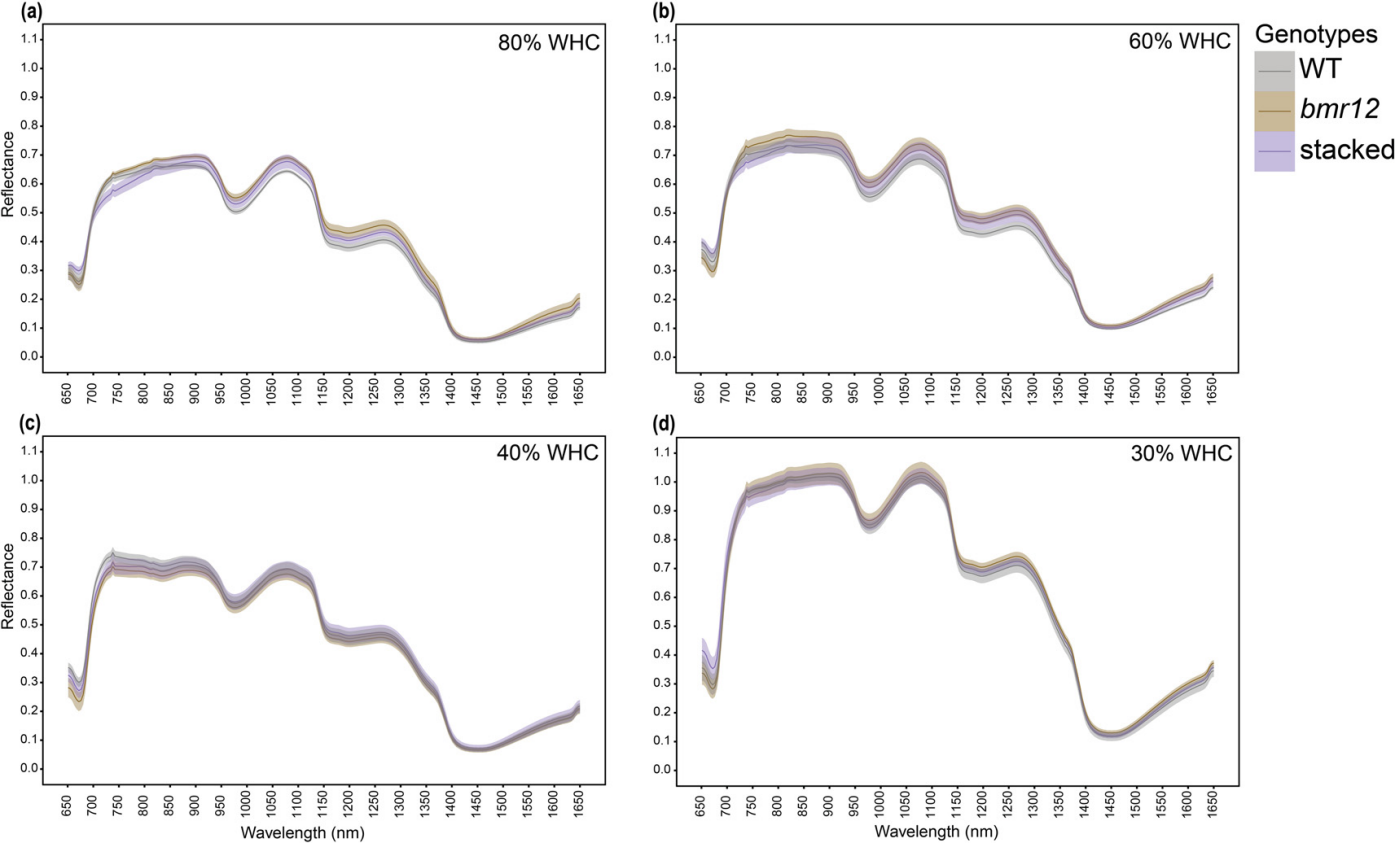
The averaged stalk spectral signatures of genotypes at HSI-4 (89 DAT) under different water treatments and fresh imaging conditions in the spectral range 650–1650 nm (n = at least 3 for each group): **(a)** Spectral reflectance of genotypes at 80% WHC **(b)** Spectral reflectance of genotypes at 60% WHC **(c)** Spectral reflectance of genotypes at 40% WHC **(d)** Spectral reflectance of genotypes at 30% WHC; WHC indicates water holding capacity. The solid line represents the mean of the samples and the ribbon around represents Standard Error (SE).

**Figure 8:**
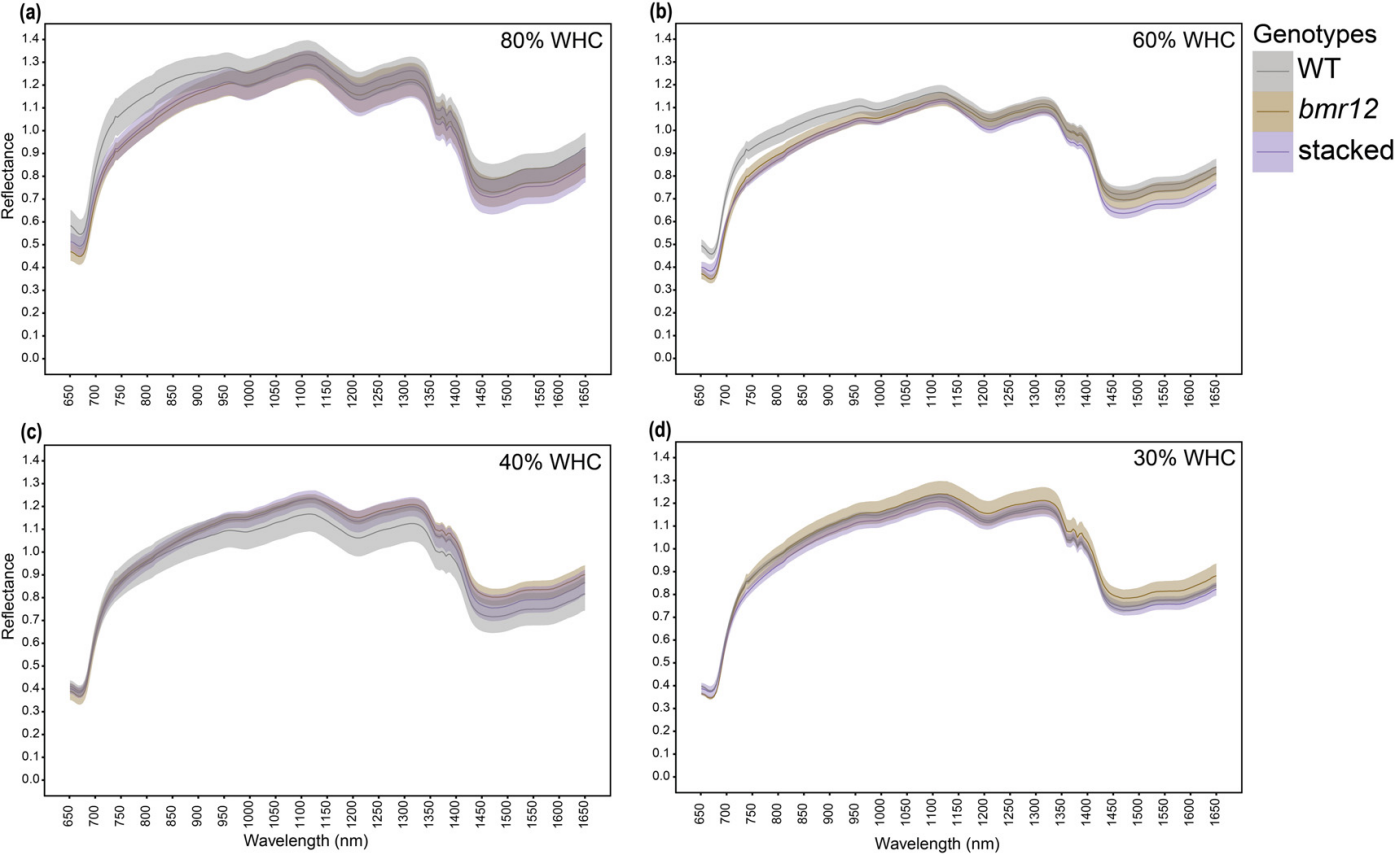
The averaged stalk spectral signatures of genotypes at HSI-4 (89 DAT) under different water treatments and oven dried imaging conditions in the spectral range 650–1650 nm (n = at least 3 for each group): **(a)** Spectral reflectance of genotypes at 80% WHC **(b)** Spectral reflectance of genotypes at 60% WHC **(c)** Spectral reflectance of genotypes at 40% WHC **(d)** Spectral reflectance of genotypes at 30% WHC; WHC indicates water holding capacity. The solid line represents the mean of the samples and the ribbon around represents Standard Error (SE).

Stalk spectral reflectance was measured for samples harvested at HSI-4 (89 DAT). For 80% WHC fresh samples (**Figure 7a**), a large separation was detected among the spectral signatures of the genotypes between 750–1350 nm, and 1550–1650 nm. A higher level of separation was observed between WT and *bmr* mutants (*bmr12* and stacked*)* as compared to between *bmr12* and stacked. The spectral trend was mostly unchanged between treatments 80% and 60% WHC (**Figures 7a and 7b**). The small changes in the spectral response of the genotypes at 60% WHC highlighted the sensitivity of the system to capture spectral differences in response to water treatment. For both 80% and 60% WHC, spectral reflectance trends among the genotypes changed around 850 nm where stacked signature crossed WT signature. Under water-deficit conditions, spectral reflectance among the genotypes differed from well-watered conditions (**Figures 7c, d**), which could be attributed to compositional changes associated with long-term exposure to water deficit conditions. Samples from 40% WHC (**Figure 7c**), had lower separation among the spectral signatures of the genotypes as compared to 80% and 60% WHC. At 40% WHC, the spectral reflectance trends among the genotypes were unique across the spectral range and the change in spectral trend observed at 850 nm for other treatments occurred around 950 nm. Unlike 40% WHC, the reflectance trends among the genotypes from 30% WHC (**Figure 7d**) were similar to those from 80% and 60% WHC. The spectral signatures of the genotypes from 30% WHC had the least separation and highest mean reflectance of all the water treatment levels; the latter was similar to the drying response of stalks (**Figure 2b**). The oven-dried stalk samples notably varied from their fresh imaged counterparts in terms of spectral trends and reflectance values (**Figure 8**). Each treatment had a unique spectral trend among the genotypes. The characteristic 1300–1350 nm range reflectance feature observed in **Figure 2** was also found in oven dried samples across the treatments. For oven-dried samples from 80% WHC (**Figure 8a**), a large separation was detected among the spectral signatures of the genotypes between 750–850 nm. As observed for fresh imaged samples, the differences between 80% WHC and 60% WHC samples for oven-dried imaging data were small (**Figure 8b**). For 80% WHC and 60% WHC oven-dried samples, the spectral reflectance based order of genotypes matched WT > *bmr12 >* stacked for lignin content estimate. At 40% WHC (**Figure 8c**), WT had the lowest mean reflectance, which was a reversed trend when compared to 80% and 60% WHC. At 30% WHC (**Figure 8d**), *bmr12* had the highest mean reflectance, and separation between the genotypes was lower as compared to other treatments. In terms of the relative reflectance trends, as the water deficit conditions were exacerbated, the mean reflectance for *bmr12* increased, whereas the reflectance for WT decreased (**Figure 8**). Across the treatments, with the exception of 30% WHC, a higher level of separation was observed between WT and *bmr* mutants (*bmr12* and stacked) as compared to between *bmr12* and stacked (**Figures 8a–c**). Although it was difficult to distinguish mutants from WT under water deficit conditions, they were easily discernable under 80% and 60% WHC for both the fresh and oven-dried samples. Irrespective of the treatment and water content of the imaged samples, spectral signatures of the genotypes maintained separation between 600–900 nm, and around 1200 nm at which lignin had strong absorption features, as observed in **Figure 2** and previously reported (Curran, 1989). Overall, the results showed that *bmr* mutants have distinct spectral signatures compared to WT under both fresh and dried imaging conditions. The stalk tissue-based spectral differences among the genotypes resolved better than foliar tissue and are more suitable to correlate with biochemical differences among the genotypes.

**Figure 9:**
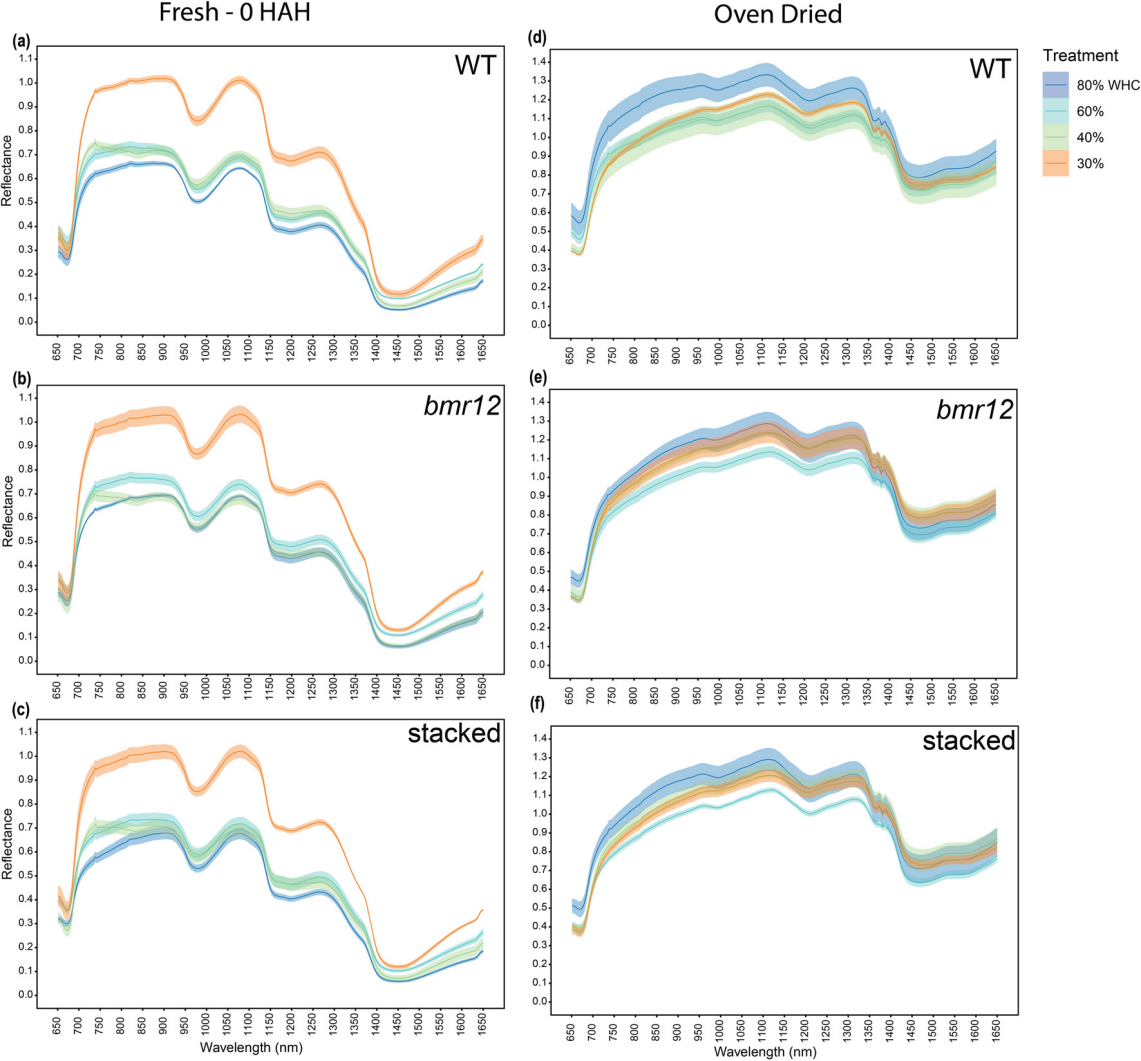
The averaged stalk spectral signatures from different water treatments for each genotype at HSI-4 (89 DAT) under different imaging conditions in the spectral range 650–1650 nm (n = at least 3 for each group): **(a)** Spectral reflectance at different treatments for WT fresh imaged samples **(b)** Spectral reflectance at different treatments for *bmr12* fresh imaged samples **(c)** Spectral reflectance at different treatments for stacked fresh imaged samples **(d)** Spectral reflectance at different treatments for WT samples imaged after oven drying **(e)** Spectral reflectance at different treatments for *bmr12* samples imaged after oven drying **(f)** Spectral reflectance at different treatments for stacked samples imaged after oven drying; WHC indicates water holding capacity. The solid line represents the mean of the samples and the ribbon around represents Standard Error (SE).

Across growth stages and tissue types, the spectral trends were treatment dependent and had clear differences between fresh and oven-dried samples. To further our understanding of the effect of water availability on the spectral signatures of the genotypes, the response of individual genotypes under the different treatment levels was plotted. For fresh imaged samples (**Figures 9a–c**), 30% WHC had the highest mean reflectance across genotypes. There was a genotype-dependent response of spectral signatures observed at 60% and 40% WHC, whereas 80% WHC had the lowest mean reflectance for all genotypes. For fresh imaged samples, WT samples from 60% and 40% WHC overlapped for most of the spectrum except between 700–800 nm and 1400–1650 nm (**Figure 9a**). For *bmr12* (**Figure 9b**), the mean reflectance for samples from 60% WHC was higher than 40% WHC for most of the spectrum. The spectral reflectance for *bmr12* at 40% WHC and 80% WHC overlapped for most parts of the spectrum. The spectral trends for stacked (**Figure 9c**) were more closely related to WT. The results for fresh imaged samples showed that certain regions of the spectrum contained unique spectral features that distinguished samples from the same genotype experiencing different levels of water availability. For samples imaged after oven drying (**Figures 9d–f**), the trends reversed when compared with fresh imaged samples; 80% WHC had the highest mean reflectance. The spectral response at other treatments followed a genotype-dependent trend. The spectral response of *bmr12* (**Figure 9e**) and stacked (**Figure 9f**) was more similar to each other than to WT (**Figure 9d**). For *bmr12* (**Figure 9e**), samples from 60% WHC had the lowest spectral reflectance. Spectral reflectance of *bmr12* samples from 30% WHC and 40% WHC overlapped for most of the spectrum, and 1350 nm onwards crossed 80% WHC signature. From 1350–1650 nm, the highest mean reflectance was observed for samples from 40% WHC, followed by 30%, 80% and 60% WHC. For stacked (**Figure 9f**), the spectral trends resembled *bmr12*. The spectral signatures for oven-dried samples suggested biochemical changes associated with water treatments. The spectral response for fresh imaged samples at 60% and 40% WHC showed a genotype-dependent response (**Figures 9a–c**), whereas the spectral response for oven-dried samples at 40% and 30% WHC showed a genotype-dependent response (**Figures 9d–f**). Irrespective of the genotype, the spectral signatures at 80% and 30% WHC differed the most for fresh samples (**Figures 9a–c**), whereas for oven-dried samples 80% and 60% WHC had the largest separation in their spectral signatures(**Figures 9d–f**). Overall, these results show that treatment-dependent changes in spectral trends for vegetative tissue were due to differential response of genotypes to water treatments.

### 3.4 Changes in energy concentration of stalk tissue in response to water limitation

**Figure 10:**
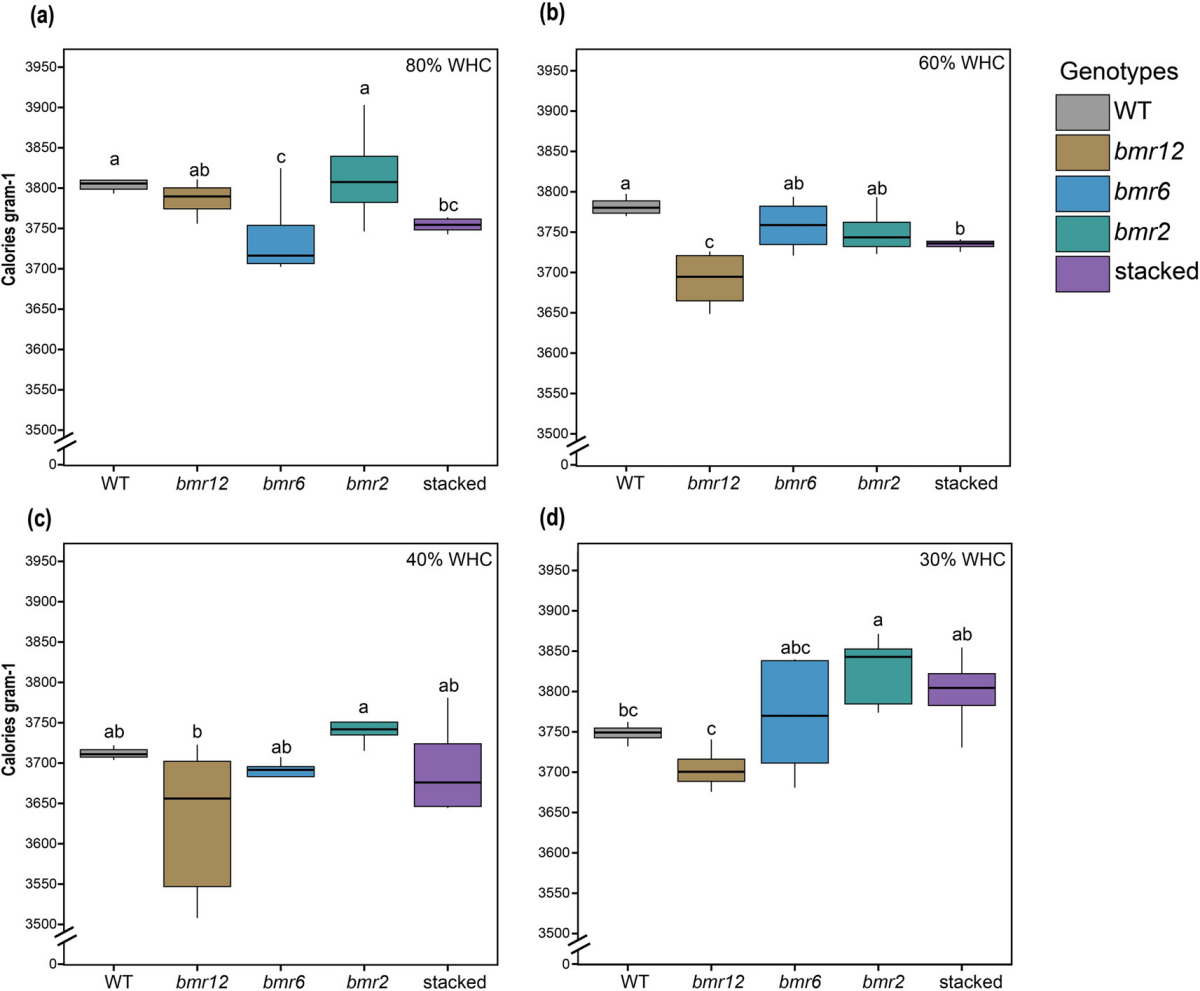
Total energy content of the imaged stalk samples from HSI-4 (89 DAT) under different water treatments (n = at least 4) **(a)** Total energy content of *bmr* mutants and WT at 80% WHC **(b)** Total energy content of *bmr* mutants and WT at 60% WHC **(c)** Total energy content of *bmr* mutants and WT at 40% WHC **(d)** Total energy content of *bmr* mutants and WT at 30% WHC; WHC indicates water holding capacity. The boxes represent the interquartile range (IQR) of the data. The horizontal line inside each box is median and whiskers extend to the minimum and maximum values within 1.5 times of IQR. Significance based on t-test with p<0.05.

To determine changes in the biochemcial composition of samples collected for HSI, oven-dried samples were used for calorimetric measurements corresponding to the oven-dried imaging material. Calorimetric measurements depicted compositional differences for WT, *bmr12*, and stacked under well-watered conditions (**Figure 10a**). At 80% WHC, WT and *bmr2* had comparable cal/g values, followed by *bmr12* and stacked, which were significantly lower than WT; *bmr6* had the lowest mean cal/g value but not significantly different from stacked. At 60% WHC (**Figure 10b**), consistent with 80% WHC results, WT had the highest energy concentration (cal/g), and all the *bmr* mutants had lower values than the WT. This finding was consistent with the observed spectral differences at the same treatment level, where *bmr12* and stacked were more distinguishable from WT when imaged fresh and after oven drying (**Figure 8b**). All genotypes except *bmr6* had lower energy concentration at 60% WHC compared to 80% WHC; the change was greater for *bmr12* (**Supplementary Figure S4**). At 40% WHC (**Figure 10c**; **Supplementary Figure S4**), a significant reduction in energy values for all genotypes was observed. The energy density for *bmr12* was the lowest, and *bmr2* was the highest, whereas WT, *bmr6*, and stacked had comparable values. At 30% WHC (**Figure 10d**), the trend reversed for all genotypes. After a gradual reduction in energy density values from 80% to 40% WHC, genotypes generally had higher energy concentration at 30% WHC (**Supplementary Figure S4**). At 30% WHC, calorimetric values were comparable for *bmr6, bmr2*, and stacked, followed by WT and *bmr12*. Plants at 30% WHC showed symptoms of severe water limitation (**Supplementary Figure S2**). The phenotypes ranged from senescence, leaf rolling and stunted growth. The energy values for *bmr6, bmr2* and stacked at 30% WHC were comparable to 80% WHC. Interestingly, as observed in **Figures 9d–f**, the spectral signatures for 80% WHC and 30% WHC were more similar compared to other treatments.

Genotypes displayed a small decrease in energy value at 60% WHC, which may explain the differences in spectral signatures among samples from 80% and 60% WHC (**Figure 8**). Larger changes in cal/g and spectral trends were detected under water deficit conditions (**Figures 7**, **8**). As observed in **Supplementary Figure S4**, *bmr12* tissues showed the greatest changes in energy value in response to water-deficit conditions, whereas *bmr2* and *bmr6* tissues were more impervious to this water stress regime. Each genotype varied in direction and magnitude of change in cal/g, which could potentially explain the genotype-dependent response of spectral signatures under this water stress regime (**Figure 9**). These results further support the differential genotypic sensitivity to water stress.

### 3.5 Classification and clustering

**Figure 11:**
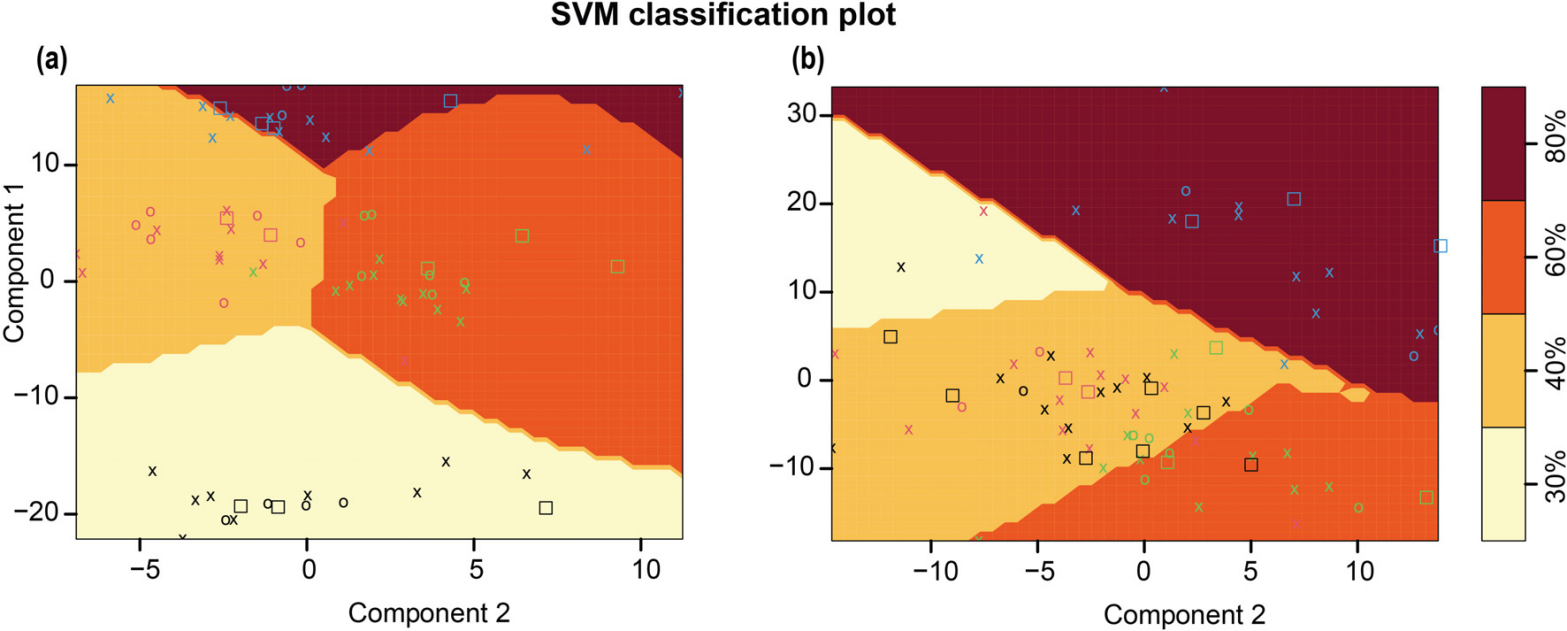
Support Vector Machine (SVM) classification for spectral data from different treatments along with the shaded decision boundaries. **(a)** SVM classifier for fresh imaging data **(b)** SVM classifier for oven dried imaging data. For a given treatment, training data points are denoted by O’s, support vectors are denoted by X’s, and test data points are denoted by squares. O's, X's, and squares for each treatment class are denoted by the same color, 80% (blue), 60% (green), 40% (red), and 30% (black).

To test the possibility of classifying samples into different treatment levels based on spectral signals, a C-classification SVM classifier with a radial kernel was used. As observed in **Figure 11a**, the SVM classifier effectively partitioned observations from fresh imaging data into treatments, based on spectral data. The SVM classifier achieved 100% accuracy on the training and test data with components from the final selected PLSR model as input (**Supplementary Table S1**). Clustering was then extended to oven-dried imaging data. The observations did not cluster naturally, and the gap statistic did not recognize clustering as beneficial. However, with the final PLSR components as input, SVM classification yielded near perfect accuracy in the training data and the test data (**Figure 11b**; **Supplementary Table S2**). The algorithm did not effectively differentiate spectral signatures from 40% WHC. SVM classification was more effective for fresh imaging data; the spectral changes induced in response to water limitation were useful to differentiate plants experiencing water-deficit conditions. This analysis provided novel insight into the spectral response of sorghum to water-deficit conditions. Although the treatment identifiers were not included as part of the predictive mechanism in the training of the PLSR model, clustering and classification using the PLSR components show that PLSR components encode treatment information. This exploratory analysis based on the spectral data, showed that the PLSR components are accurate for treatment classification.

### 3.6 Modeling for energy concentration using full spectral range

Statistical models including partial least squares regression (PLSR), support vector machine (SVM) and random forest (RF) were applied to establish the quantitative relationship among the extracted reflectance spectral data within the full spectral range of 242 wavelength bands (predictors) and the corresponding calorimetric data represented by 60 training and 16 test entries (response). The models were then validated using an external validation set to estimate the actual predictive ability. **Table 1** shows the results of the calibration, cross-validation, and prediction statistics of the models. As summarized in **Table 1A** for fresh imaging data, the PLSR model had a RMSE_train_ of 35.76, a r_test_ of 0.4697 and RMSE_test_ of 50.13 in prediction. When models were built using RF, the model obtained a RMSE_train_ of 20.64 in training, a r_test_ of 0.206 and RMSE_test_ 54.51 in prediction. The SVM model had least accuracy with RMSE_train_ of 49.32 in training, a r_test_ of 0.249 and RMSE_test_ 54.88 in prediction. The PLSR model outperformed other models.

**Table 1:** Cross-validation and test results of using full spectrum to predict cal/g with Partial Least Squares Regression (PLSR), Random Forest (RF), and Support Vector Machine (SVM).

Table 1A	Error metrics for fresh imaging data				
Method	RMSE_train_	RMSE_CV_	RMSE_test_	r_cv_	r_test_
PLSR	35.76	43.67	50.13	0.517	0.470
RF	20.64	43.94	54.51	0.448	0.206
SVM	49.32	51.2	54.88	0.106	0.249
Table 1B	Error metrics for oven dried imaging data				
Method	RMSE_train_	RMSE_CV_	RMSE_test_	r_cv_	r_test_
PLSR	29.34	39.05	44.33	0.644	0.602
RF	24.22	51.22	56.607	0.065	0.255
SVM	49.83	51.29	55.33	0.087	0.170

The calorimetric measurements were conducted on oven-dried samples. Therefore, oven-dried spectral data-based modeling was performed with the hypothesis that prediction should be better for oven-dried data as compared to fresh imaging data. As shown in **Table 1B**, the oven-dried prediction models outperformed fresh imaging-based models. The PLSR model had a RMSE_train_ of 29.34 in training, a r_test_ of 0.602 and RMSE_test_ 44.33 in prediction. When models were built using RF, the model obtained a RMSE_train_ of 24.22 in training, a r_test_ of 0.255 and RMSE_test_ 56.607 in prediction. The SVM model had least accuracy with RMSE_train_ of 49.83 in training, a r_test_ of 0.170, and RMSE_test_ 55.33 in prediction. As observed for fresh imaging data, the PLSR model outperformed other models. RF and SVM functioned better for fresh imaging data compared to oven-dried data; however, overall, they were not accurate enough for predictive use. PLSR outperformed all the models. Although fresh imaging data was useful for predicting cal/g based on PLSR, the accuracy of the PLSR model was significantly higher for the oven-dried data, as expected (**Supplementary Figure S5**).

### 3.7 Modeling energy concentration using optimal wavelengths

Although PLSR based predictions using full spectral range of 242 wavelength bands (predictors) was efficient, an equation with smaller group of predictors can be more tractable. To develop an equation using relevant predictive wavelengths, the LASSO approach was used to obtain a sparse prediction equation. LASSO regression allowed us to generate a sparse model with non-zero coefficients for a small subset of wavelengths. As shown in **Table 2A**, the LASSO model had marginally lower prediction accuracy than PLSR for fresh imaging data, whereas its prediction accuracy for oven-dried data was comparable to that of PLSR (**Supplementary Figure S6**). For fresh imaging data, the LASSO model had a RMSE_train_ of 37.366 in training, a r_test_ of 0.389 and RMSE_test_ 51.83 in prediction. For oven dried imaging data, the LASSO model had a RMSE_train_ of 28.325 in training, a r_test_ of 0.604 and RMSE_test_ 43.675 in prediction. The LASSO model for fresh imaging data used 14 wavelengths with non-zero coefficients to establish predictions (**Table 2B**). The reported wavelengths had the strongest predictive power for calorimetric content of the samples compared to other wavelengths for fresh imaging data. For samples imaged after oven drying, 22 wavelengths had high predictive power for calorimetric content of the samples (**Table 2B**). Most differences among the spectral signatures of the genotypes were detected between 600–900 nm and around 1200 nm (**Figures 7**, **8**). The wavelength vectors with non-zero coefficients primarily resolved to the same spectral range. These results demonstrated that the differences in spectral signatures were based on changes in biochemical composition and could gauge calorimetric measurements.

**Table 2:** Cross-validation and test results of using optimal wavelengths based on LASSO model to predict cal/g.

Table 2A	Error metrics for LASSO model				
Method	RMSE_train_	RMSE_CV_	RMSE_test_	r_cv_	r_test_
Fresh	37.366	44.748	51.839	0.449	0.389
Oven Dried	28.326	44.781	43.676	0.505	0.604
Table 2B	Wavelengths with non-zero coefficients used by LASSO model to establish prediction				
Dataset	Wavelength (nm)	Coefficient			
Fresh	651.095	75.180			
Fresh	671.82	-2151.522			
Fresh	688.4	2190.856			
Fresh	729.851	-579.684			
Fresh	829.331	400.314			
Fresh	999.276	-324.228			
Fresh	1020	-968.309			
Fresh	1181.66	-1167.202			
Fresh	1185.8	-156.534			
Fresh	1289.43	0.062			
Fresh	1293.57	33.131			
Fresh	1301.86	155.861			
Fresh	1306.01	314.797			
Fresh	1310.15	2049.670			
Oven Dried	667.675	-1238.971			
Oven Dried	671.82	-858.982			
Oven Dried	684.255	789.914			
Oven Dried	688.4	2311.060			
Oven Dried	709.126	-1862.389			
Oven Dried	738.141	139.271			
Oven Dried	779.591	0.009			
Oven Dried	783.736	1106.862			
Oven Dried	866.636	10.645			
Oven Dried	1136.06	-2188.964			
Oven Dried	1223.11	2234.857			
Oven Dried	1231.4	134.342			
Oven Dried	1359.89	-986.749			
Oven Dried	1372.33	1783.041			
Oven Dried	1397.2	6.115			
Oven Dried	1401.34	871.405			
Oven Dried	1430.36	-46.357			
Oven Dried	1434.5	-5421.559			
Oven Dried	1438.65	-1628.416			
Oven Dried	1484.24	233.635			
Oven Dried	1492.53	6281.460			
Oven Dried	1650.04	-1819.286			

### 3.8 Modeling for relative water content using full spectral range

RWC was recorded for drying samples at regular intervals of fresh (0), 6, 12, 24, 48, 72, 96, 103, 120 HAH and oven-dried samples. This avoided double counting as the samples were imaged at every time point and had an updated RWC value for every recording. For every treatment with at least 18 samples and 10 time points, a total of at least 180 data points were available, which made it possible to generate treatment-based prediction equations and provided a comprehensive data frame to conduct prediction modeling. Calibration algorithms of PLSR, RF, and SVM were applied to establish the quantitative relationship between the extracted reflectance spectral data within the full spectral range of 242 wavelength bands (predictors) and the corresponding dependent RWC (predicted). **Table 3** shows the results of the calibration, cross-validation, and prediction statistical parameters of the models. As summarized in **Table 3A** for overall prediction (not including treatments as a factor) modeling, all models had high accuracy. The PLSR model had a RMSE_train_ of 0.330 in training, a r_test_ of 0.985 and RMSE_test_ 0.240 in prediction. When models were built using RF, the model obtained RMSE_train_ of 0.188 in training, a r_test_ of 0.975, and RMSE_test_ 0.302 in prediction. Meanwhile, the SVM model was least accurate with RMSE_train_ of 1.121 in training, a r_test_ of 0.867, and RMSE_test_ 0.702 in prediction. **Table 3B** summarizes the statistical parameters for treatment-based prediction models. All models predicted RWC under all treatments accurately. **Supplementary Figure S7** presents the scatter plots of measured versus predicted values of the PLSR and RF models.

**Table 3:** Cross-validation and test results of using full spectrum to predict Relative Water Content (RWC) with Partial Least Squares Regression (PLSR), Random Forest (RF), and Support Vector Machine (SVM).

Table 3A	Error metrics for overall data					
Method	RMSE_train_	RMSE_CV_	RMSE_test_	r_cv_	r_test_	
PLSR	0.330	0.359	0.240	0.969	0.985	
RF	0.188	0.424	0.302	0.957	0.975	
SVM	1.121	1.003	0.702	0.791	0.867	
Table 3B	Treatment-wise error metrics using treatment as factor					
Treatment	Method	RMSE_train_	RMSE_CV_	RMSE_test_	r_cv_	r_test_
80% WHC	PLSR	0.276	0.328	0.218	0.959	0.981
80% WHC	RF	0.133	0.287	0.228	0.969	0.973
80% WHC	SVM	1.091	1.057	0.828	0.627	0.651
60% WHC	PLSR	0.184	0.259	0.243	0.983	0.989
60% WHC	RF	0.183	0.426	0.434	0.954	0.960
60% WHC	SVM	1.376	1.320	1.467	0.487	0.691
40% WHC	PLSR	0.297	0.380	0.575	0.963	0.952
40% WHC	RF	0.169	0.412	0.687	0.956	0.936
40% WHC	SVM	1.345	1.185	1.406	0.641	0.764
30% WHC	PLSR	0.283	0.324	0.377	0.976	0.975
30% WHC	RF	0.149	0.294	0.420	0.981	0.970
30% WHC	SVM	1.425	1.349	1.229	0.658	0.666

### 3.9 Prediction accuracies between half and full section imaging are comparable

To minimize the potential for error due to shadowing between the adjacent split stalk sections, we used half-sections (**Figure 1**). As shown in **Supplementary Table S3**, there was no significant improvement in prediction accuracy for cal/g. **Supplementary Table S4** summarizes no significant improvement in prediction accuracy for RWC. The scatter plots of measured versus predicted value of the PLSR and RF models are shown for cal/g (**Supplementary Figure S8**) and for RWC (**Supplementary Figure S7**). These results suggest that shadow effect between the adjacent split stalk sections was not an issue, and confirmed the reliability of this approach. The comparable predictions from both methods emphasize that the half-section results served as a technical replicate for the full-section method.

## 4 Discussion

In this study, we aimed to link known biochemical composition differences with their spectral signatures under four water treatments. The HSI setup effectively differentiated among different classes of macromolecules, and the system was able to distinguish the subclasses of carbohydrates, including starch and cellulose (**Figure 2a**), reflecting the sensitivity of the technique. Starch has α(1→4) glycosidic bonds, whereas cellulose has β(1→4) glycosidic bonds where alternate beta-glucose monomers are oriented 180° to each other (Richardson and Gorton, 2003). The α(1→6) glycosidic bonds establish branching in starch, whereas linear cellulose establishes interchain hydrogen bonding with parallel chains (Richardson and Gorton, 2003). The differentiating spectral features between starch and cellulose reported in this study could correspond to differences in the orientation of alternate monomers or branching. The spectral differences observed in this study were larger between different classes as compared to between different subclasses. To test the reliability of the setup, different derivatives of the same casein protein sample were examined, casein acid hydrolysate and casein enzymatic hydrolysate, which all had the same spectral signature (**Figure 2a**). Lignin has a unique spectral signature, which could be a feature of its ether bonds and aromatic rings. The absorption wavelengths associated with various organic compounds based on NIRS (Curran, 1989), fall into the observed absorption range using HSI (**Figure 2a**). This served as a validation of the technique for its reliability in detecting true biochemical differences. The tested standards shared the same absorption feature between 1350–1400 nm, validating that biological compounds absorb light at similar wavelengths (Curran, 1989). It also suggests that this feature could be indicative of a specific chemical bond present in all the samples, which, from the structure of these compounds, could be covalent bonds such as C-C and C-H bonds. We have ruled out the C-O bond because it is also shared by urea, which has a unique spectral signature lacking a strong 1350–1400 nm feature. The unique spectral signature of urea could be a combined feature of amide bonds, high N:C ratio, and planar geometry (Vaughan and Donohue, 1952). The spectral signature of standards (**Figure 2a**) could be further expanded and developed into a library where the spectral signatures of plant tissue samples could be screened for potential matches. Percentage identity indicators could be provided based on matching features of reflectance (crest) and absorption (trough) across the spectral range. We propose the possibility of developing an algorithm for local alignment of the spectral signatures of a plant tissue sample, based on percent identity or similarity score in comparison with the signature of standards of organic compounds. The technique suggests the next step in narrowing down the biological basis of spectral differences between plant samples.

To identify plant-specific spectral features for differences in biochemical composition, the sorghum *bmr* mutants impaired in the monolignol biosynthesis were used as a model. For varying level of dehydration of stalks (**Figure 2b**), there is a linear relationship between sample dehydration and reflectance. The observed trend, shared by the tested standards between 1350–1400 nm, was also detected for oven dried plant tissue (**Figure 2**) across tissue types and growth stages. Lack of this trend in fresh tissue is likely due to water masking the biochemical spectral signatures. The differences targeted in this study are based on cell wall composition/biochemical changes. The differences in spectral trends due to water limitation for fresh samples might be partly explained by varying relative water content; however, detecting differences in spectral trends for samples imaged after oven drying emphasizes biochemical differences between the samples. The information from fresh tissue imaging is useful in detecting plants experiencing water stress. There are identifying features from the spectrum that are useful for distinguishing samples from different levels of water-deficit conditions for a given genotype (**Figures 9a–c**). This example shows the potential use case scenario where fresh sample imaging is more useful than dried sample imaging. The SVM classifier effectively separates both training and test samples into respective treatment classes based on spectral signatures (**Figure 11**). This setup was able to identify plants experiencing 60% WHC, when symptoms of limited water availability were not visible to the human eye, which emphasizes the potential of this technique to sense plant stress at the initial stages. Fresh and dried imaging data from the same samples yield unique information for each category. Therefore, to explore spectral differences between samples using HSI, studies should, where feasible, incorporate both fresh and oven-dried samples into their experimental designs. The ability of the system to not only detect differences between different genotypes under varying treatment levels but also differentiate a given genotype under varying water-deficit treatments suggests that it is a robust technology to identify compositional differences and changes induced by water-deficit conditions.

In this study, genotypic spectral differences were detected using foliar tissue (leaf blade and isolated midrib) samples. Different growth conditions and growth stages alter the spectral signature of leaf samples (**Figures 3**–**5**). There we cannot discount the possibility of interference in the leaf or midrib spectral signatures from pigments contributing to the brown midrib color (**Figure 6**). The changing spectral trends among the genotypes between growth stages and treatments emphasize the fact that HSI-based feature detection depends on biochemical composition during growth (Kamstra et al., 1958; McBee and Miller, 1993) or stress exposure (Emerson et al., 2014; Perrier et al., 2017). Our HSI was able to record changes in spectral signatures during the early stages of water limitation (**Figure 3**) when the effects are not visible to the human eye. We detected features that potentially link to lignin content, but considering large sections of the spectrum with overlapping means and SE for the genotypes (**Figures 3**–**6**), the accuracy of the statistical regression models may be relatively low compared to direct chemical analyses of compounds. This could be due to relatively low concentration of lignin in foliar tissue (McBee and Miller, 1993). Overall, midribs sampled at the end of the vegetative stage were the more suitable foliar tissue to establish spectral trends based on biochemical differences.

Stalk tissue contributes most to plant stover (stalks and leaves) in terms of biomass. Stalks are also enriched in lignin due to the elaborate vascular bundles contained inside (McBee and Miller, 1993; Hatfield et al., 1999). Based on larger separation in the means and SE among the genotypes, stalk tissue was the preferred sample for detecting lignin-based differences between the genotypes and treatments. Water limitation alters the hyperspectral signature (Emerson et al., 2014; Perrier et al., 2017). Our results show that sorghum *bmr* mutants have altered spectral response under water limitation (**Figures 9d–f**), which likely reflects differential sensitivity to water limitation-induced biochemical changes.

Lignin has the highest energy density of all biochemical components of a plant cell wall (Novaes et al., 2010) and a high degree of correlation with calorimetric (energy) data (Gardner et al., 2015). Calorimetric data reflect changes in the overall biochemical composition of the plant tissue and also serves as a proxy for lignin content. A representative 15 cm stalk tissue above the crown was sufficient to detect calorimetric differences among the genotypes. Machine learning models were developed using spectral data from the stalk tissue to effectively determine the energy value of test data. PLSR performed better than other models because of its ability to effectively handle multicollinearity in our study, which is a feature of hyperspectral data (Sun, 2010). Oven-dried tissue is most suitable for estimating calorimetric content based on spectral information, which is logical given calorimetric estimations were based on data from oven-dried samples. The models trained using stalk tissue are a useful tool to estimate the energy value of samples using a lot less tissue, simplifying the sample processing. This study shows that the features evaluated through the HSI system could be used to characterize forages with altered lignin content. The most effective calibration models use a selection of optimal wavelengths, defined by regression coefficient analysis, to reduce the computational burden of this multivariate analysis (Liu et al., 2014). Equations using fewer wavelengths address the same biological question in a shorter period and requires lower computational power (Liu et al., 2014). Based on LASSO, this study presented 14 wavelengths for fresh imaging data and 22 wavelengths for oven-dried imaging data (**Table 2B**). The reported wavelengths have the strongest predictive power for the calorimetric content of the samples compared to other wavelengths. These wavelengths should be further tested for improving prediction accuracy. The methods from this study can be useful for large breeding programs because energy density measurements per sample could be conducted on a representative 15 cm stalk tissue without time and labor intensive sample grinding and bomb calorimetry.

## 5 Conclusion

This study presents a rapid, accurate, and alternative gross energy density measurement technique for sorghum stalks. Our results indicate the robustness of HSI for chemical sensing, wavelength range of strong absorption features for major organic compounds included absorption wavelengths previously reported using NIRS. Stalk tissues were most suitable for resolving spectral differences between genotypes. The spectral response of the genotypes was treatment dependent associated well with the calorimetric measurements. Prediction models indicated that fresh imaging is more useful to differentiate plants experiencing water-deficit conditions whereas oven dried data performed better for calorimetric estimations from the spectral data. This highlighted the importance of using tissues with varying hydrations because there were independent useful inferences from oven dried and fresh imaging data. LASSO regression generated wavelengths with high predictive power for calorimetric content of the samples. Since calorimetric measurements were used as a proxy for lignin, same subset of wavelength should have high predictive power for lignin content estimations. The wavelengths reported in this study can further broaden the applications of high-throughput energy density screening as it would enable the use of less expensive multispectral camera systems with limited spectral bands.

## 6 Limitations and future directions

Similar to most machine learning models, LASSO is limited by multicollinearity. Under situations of multicollinearity, LASSO randomly selects between highly correlated predictors, therefore a different set of wavelengths from the range of 242 predictors could also have similar prediction accuracy. This does not rule out the biological relevance of wavelengths identified in this study. Future studies will include higher number of diverse sorghum accessions to make the training data more robust and to further refine subset of wavelengths for energy density estimations from spectral data.

## Data Availability

The raw data supporting the conclusions of this article will be made available by the authors, without undue reservation.

## Supplementary material

Supplementary Figure 1 Experimental design based on a Randomized Complete Block Design (RCBD).

Supplementary Figure 2 Greenhouse setup of the experimental design.

Supplementary Figure 3 Uniform drying-based spectral response of stalk samples from 80% WHC showing relationship between tissue hydration and spectral response. **(A)** WT **(B)** *bmr2* **(C)** *bmr6* **(D)** stacked; HAH indicates hours after harvesting. The solid line represents the mean of the samples and the ribbon around represents Standard Error (SE).

Supplementary Figure 4 Total energy content of the imaged stalk samples from HSI-4 (89 DAT), genotype response under different water treatments (n = at least 4) **(A)** Total energy content of WT under different water treatments **(B)** Total energy content of *bmr12* under different water treatments **(C)** Total energy content of *bmr6* under different water treatments **(D)** Total energy content of *bmr2* under different water treatments **(E)** Total energy content of stacked under different water treatments; WHC indicates water holding capacity. The boxes represent the interquartile range (IQR) of the data. The horizontal line inside each box is median and whiskers extend to the minimum and maximum values within 1.5 times of IQR. Significance based on t-test with p<0.05.

Supplementary Figure 5 Scatterplot of the lab-measured value vs. the image-predicted value of cal/g based on the full spectrum for at least n = 75. The data were split into a training and a test set consisting of 60 and 16 observations (75/25 split), respectively. The cross validation set is denoted by dot and test data set by triangle. Grey line represents the regression line for cross validation set, red line represents the test fit regression line, and the dotted line represents 1:1 regression fit. **(A)** Partial Least Squares Regression (PLSR) for fresh imaging data **(B)** Random Forest (RF) for fresh imaging data **(C)** Partial Least Squares Regression (PLSR) for oven dried imaging data **(D)** Random Forest (RF) for oven dried imaging data. The error metrics are available in **Table 1**.

Supplementary Figure 6 Scatterplot of the lab-measured value vs. the image-predicted value of cal/g based on the optimum wavelengths from LASSO model for at least n = 75. The data were split into a training and a test set consisting of 60 and 16 observations (75/25 split), respectively. The cross validation set is denoted by dot and test data set by triangle. Grey line represents the regression line for cross validation set, red line represents the test fit regression line, and the dotted line represents 1:1 regression fit. **(A)** Fresh imaging data **(B)** Oven dried imaging data. The error metrics are available in **Table 2**.

Supplementary Figure 7 Scatterplot of the lab-measured value vs. the image-predicted value of Relative Water Content (RWC) based on the full spectrum for at least n = 660. The data were split into a training and a test set consisting of 530 and 132 observations (80/20 split), respectively. The cross validation set is denoted by dot and test data set by triangle. Grey line represents the regression line for cross validation set, red line represents the test fit regression line, and the dotted line represents 1:1 regression fit. **(A)** Partial Least Squares Regression (PLSR) for full section imaging **(B)** Random Forest (RF) for full section imaging **(C)** Partial Least Squares Regression (PLSR) for half section imaging **(D)** Random Forest (RF) for half section imaging. The error metrics are available in **Table 3** and **S4**.

Supplementary Figure 8 Scatterplot of the lab-measured value vs. the image-predicted value of cal/g based on the full spectrum for half section results with at least n = 75. The data were split into a training and a test set consisting of 60 and 16 observations (75/25 split), respectively. The cross validation set is denoted by dot and test data set by triangle. Grey line represents the regression line for cross validation set, red line represents the test fit regression line, and the dotted line represents 1:1 regression fit. **(A)** Partial Least Squares Regression (PLSR) for fresh imaging data **(B)** Random Forest (RF) for fresh imaging data **(C)** Partial Least Squares Regression (PLSR) for oven dried imaging data **(D)** Random Forest (RF) for oven dried imaging data. The error metrics are available in **Supplementary Table S3**.
