# Supplementary figures and images for: Hyperspectral imaging to characterize the vegetative tissue biochemical changes in response to water deficit conditions in sorghum (Sorghum bicolor)
Source: Front Plant Sci. 2025 May 29;16:1515998. doi: 10.3389/fpls.2025.1515998 (PMC12159069; doi:10.3389/fpls.2025.1515998)

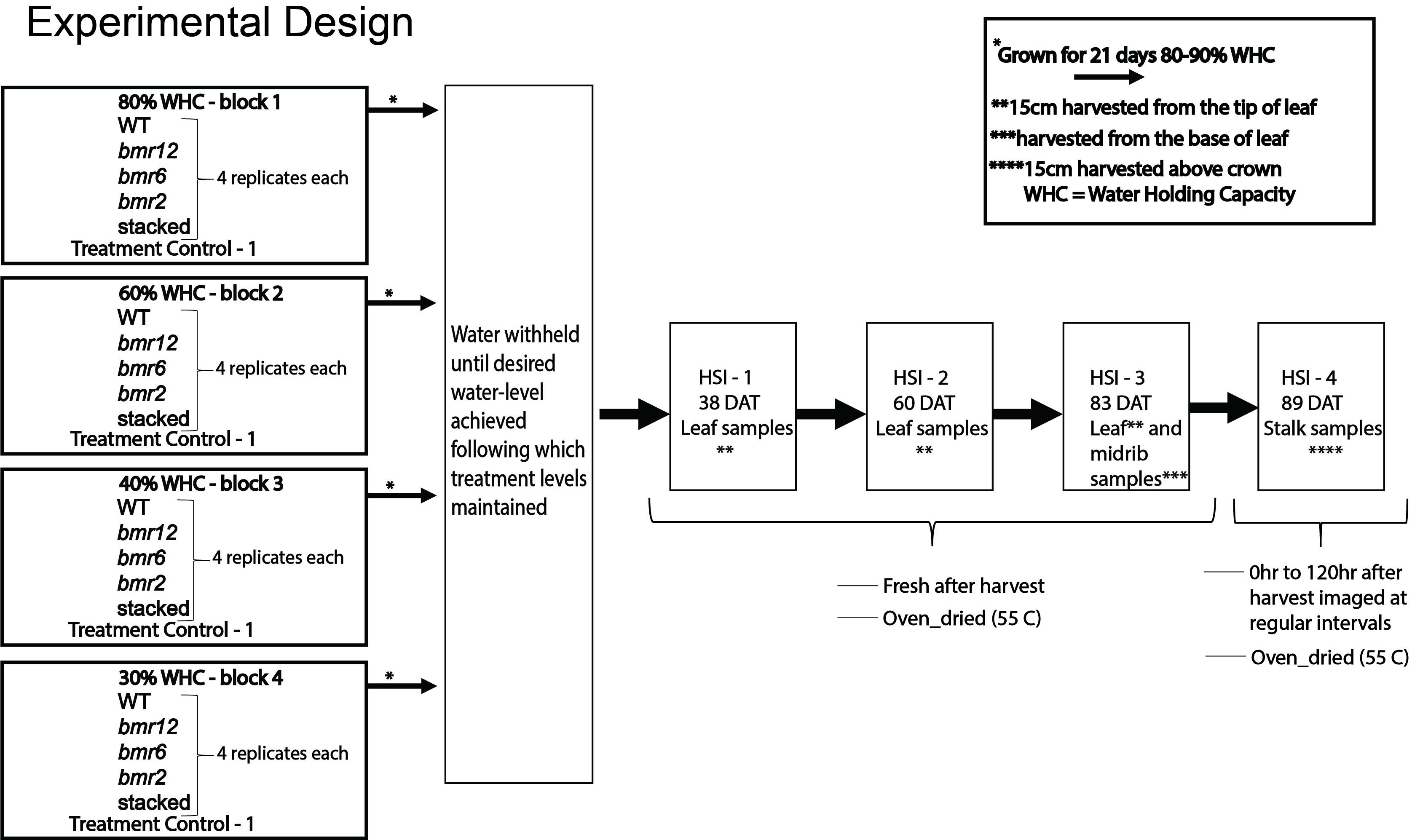

Supplement: Supplementary Figure 1 — Experimental design based on a Randomized Complete Block Design (RCBD). [file Image1.jpeg]

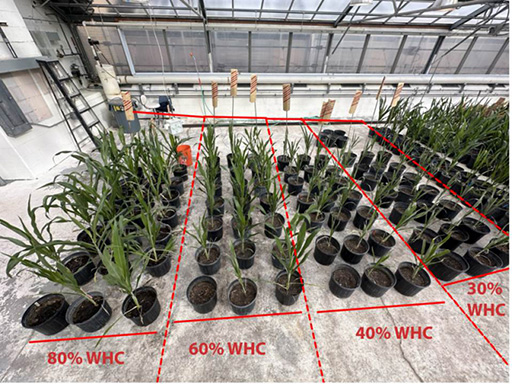

Supplement: Supplementary Figure 2 — Greenhouse setup of the experimental design. [file Image2.jpeg]

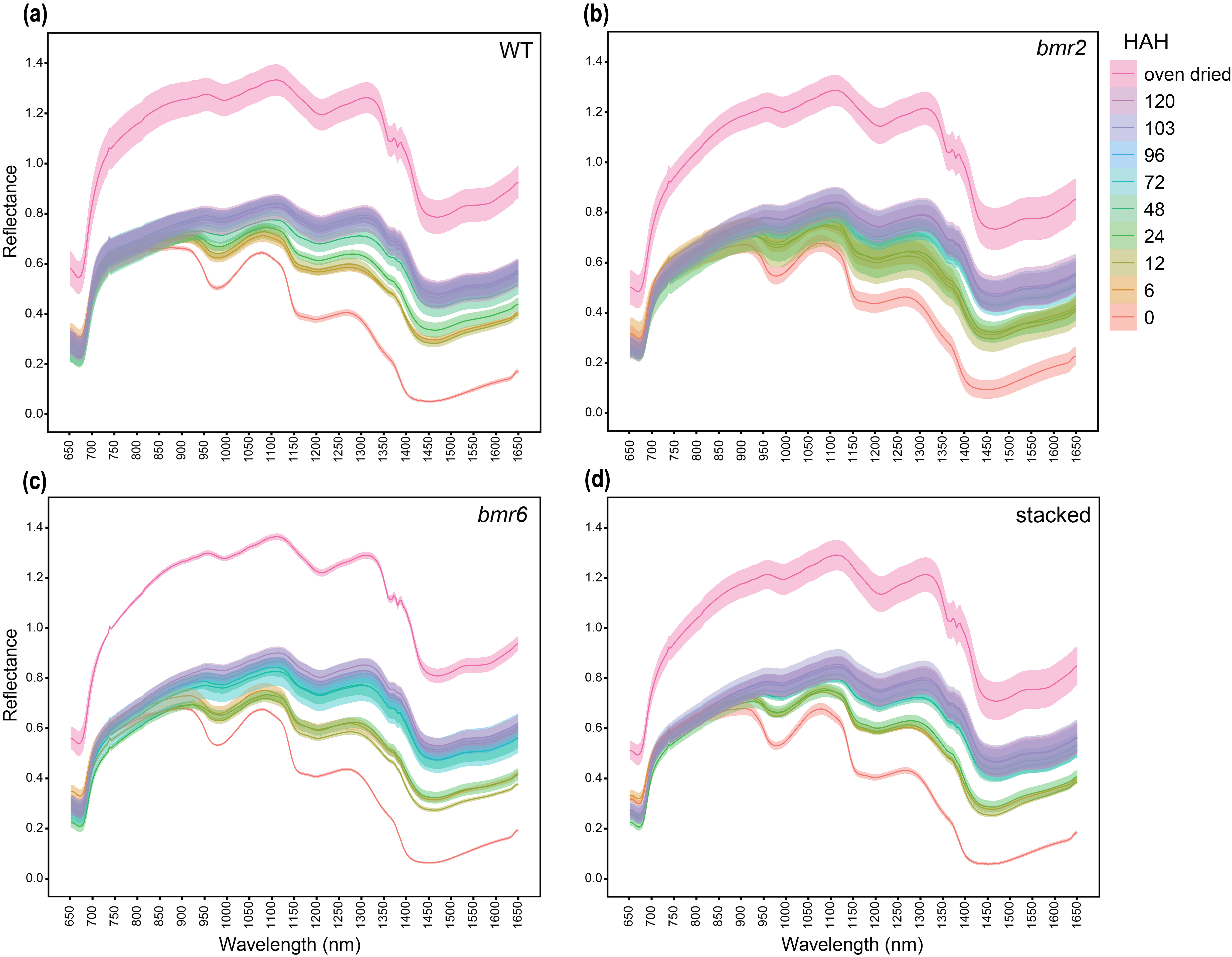

Supplement: Supplementary Figure 3 — Uniform drying-based spectral response of stalk samples from 80% WHC showing relationship between tissue hydration and spectral response. (A) WT (B) bmr2 (C) bmr6 (D) stacked; HAH indicates hours after harvesting. The solid line represents the mean of the samples and the ribbon around represents Standard Error (SE). [file Image3.jpeg]

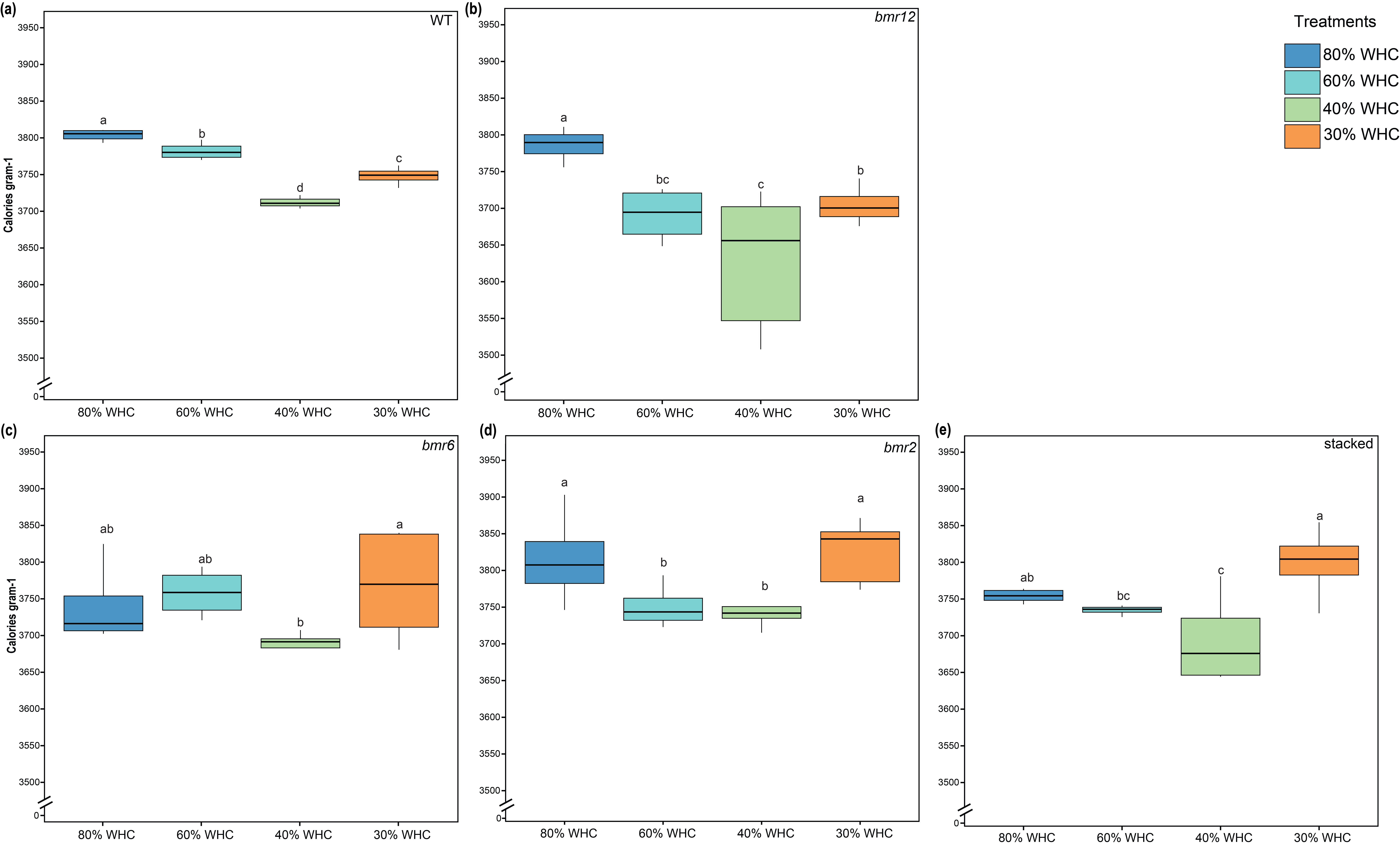

Supplement: Supplementary Figure 4 — Total energy content of the imaged stalk samples from HSI-4 (89 DAT), genotype response under different water treatments (n = at least 4) (A) Total energy content of WT under different water treatments (B) Total energy content of bmr12 under different water treatments (C) Total energy content of bmr6 under different water treatments (D) Total energy content of bmr2 under different water treatments (E) Total energy content of stacked under different water treatments; WHC indicates water holding capacity. The boxes represent the interquartile range (IQR) of the data. The horizontal line inside each box is median and whiskers extend to the minimum and maximum values within 1.5 times of IQR. Significance based on t-test with p<0.05. [file Image4.jpeg]

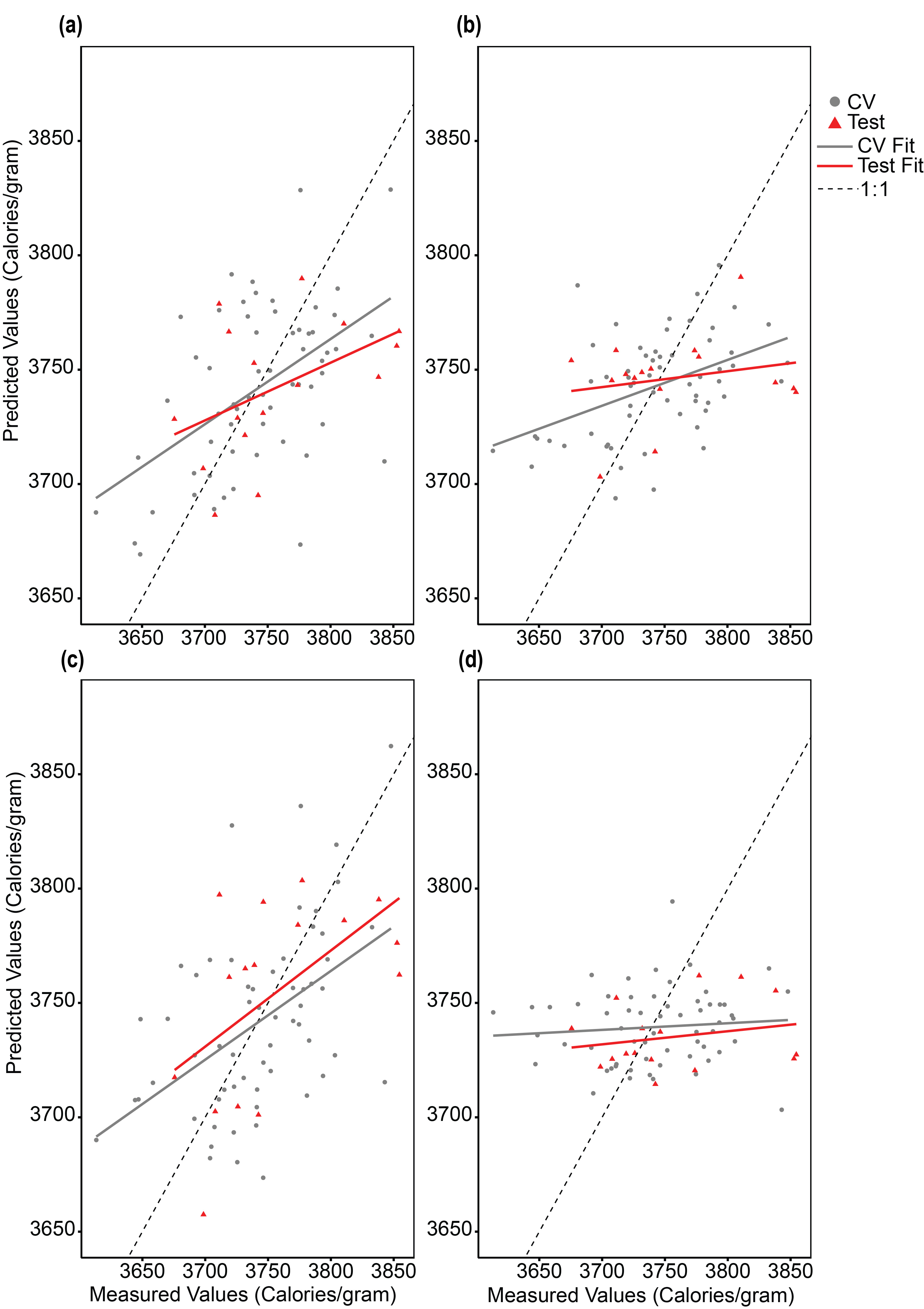

Supplement: Supplementary Figure 5 — Scatterplot of the lab-measured value vs. the image-predicted value of cal/g based on the full spectrum for at least n = 75. The data were split into a training and a test set consisting of 60 and 16 observations (75/25 split), respectively. The cross validation set is denoted by dot and test data set by triangle. Grey line represents the regression line for cross validation set, red line represents the test fit regression line, and the dotted line represents 1:1 regression fit. (A) Partial Least Squares Regression (PLSR) for fresh imaging data (B) Random Forest (RF) for fresh imaging data (C) Partial Least Squares Regression (PLSR) for oven dried imaging data (D) Random Forest (RF) for oven dried imaging data. The error metrics are available in Table 1 . [file Image5.jpeg]

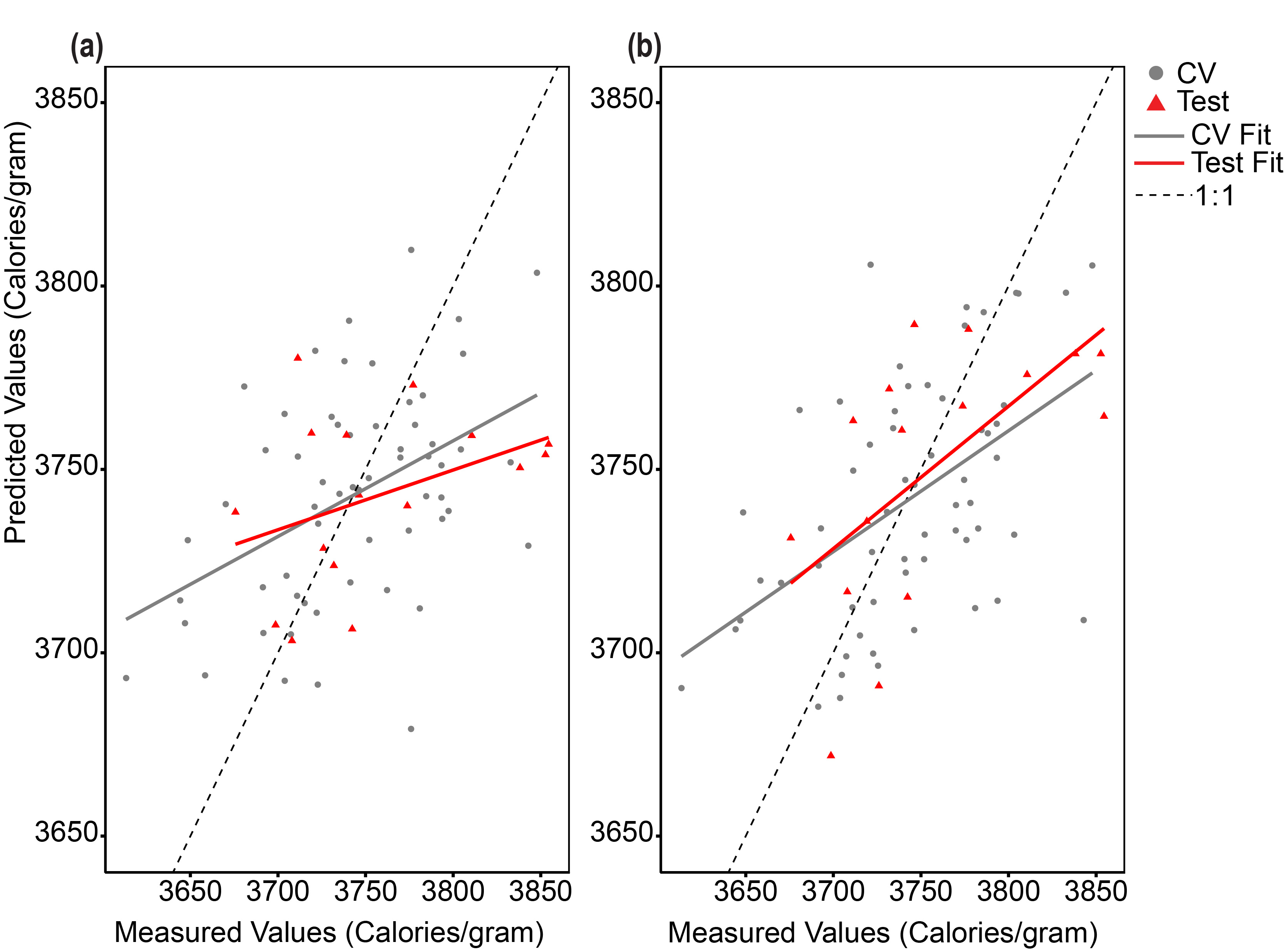

Supplement: Supplementary Figure 6 — Scatterplot of the lab-measured value vs. the image-predicted value of cal/g based on the optimum wavelengths from LASSO model for at least n = 75. The data were split into a training and a test set consisting of 60 and 16 observations (75/25 split), respectively. The cross validation set is denoted by dot and test data set by triangle. Grey line represents the regression line for cross validation set, red line represents the test fit regression line, and the dotted line represents 1:1 regression fit. (A) Fresh imaging data (B) Oven dried imaging data. The error metrics are available in Table 2 . [file Image6.jpeg]

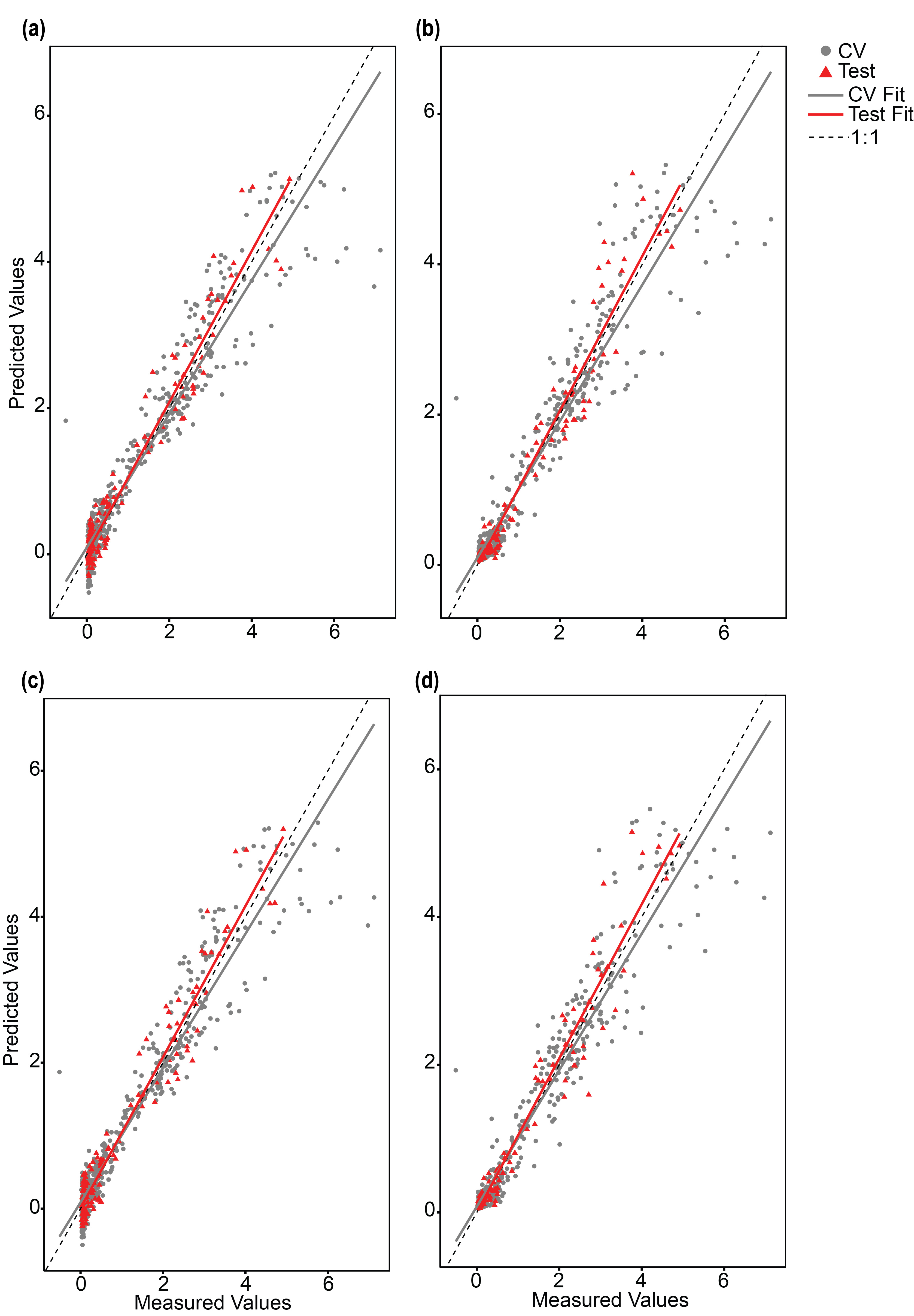

Supplement: Supplementary Figure 7 — Scatterplot of the lab-measured value vs. the image-predicted value of Relative Water Content (RWC) based on the full spectrum for at least n = 660. The data were split into a training and a test set consisting of 530 and 132 observations (80/20 split), respectively. The cross validation set is denoted by dot and test data set by triangle. Grey line represents the regression line for cross validation set, red line represents the test fit regression line, and the dotted line represents 1:1 regression fit. (A) Partial Least Squares Regression (PLSR) for full section imaging (B) Random Forest (RF) for full section imaging (C) Partial Least Squares Regression (PLSR) for half section imaging (D) Random Forest (RF) for half section imaging. The error metrics are available in Table 3 and S4 . [file Image7.jpeg]

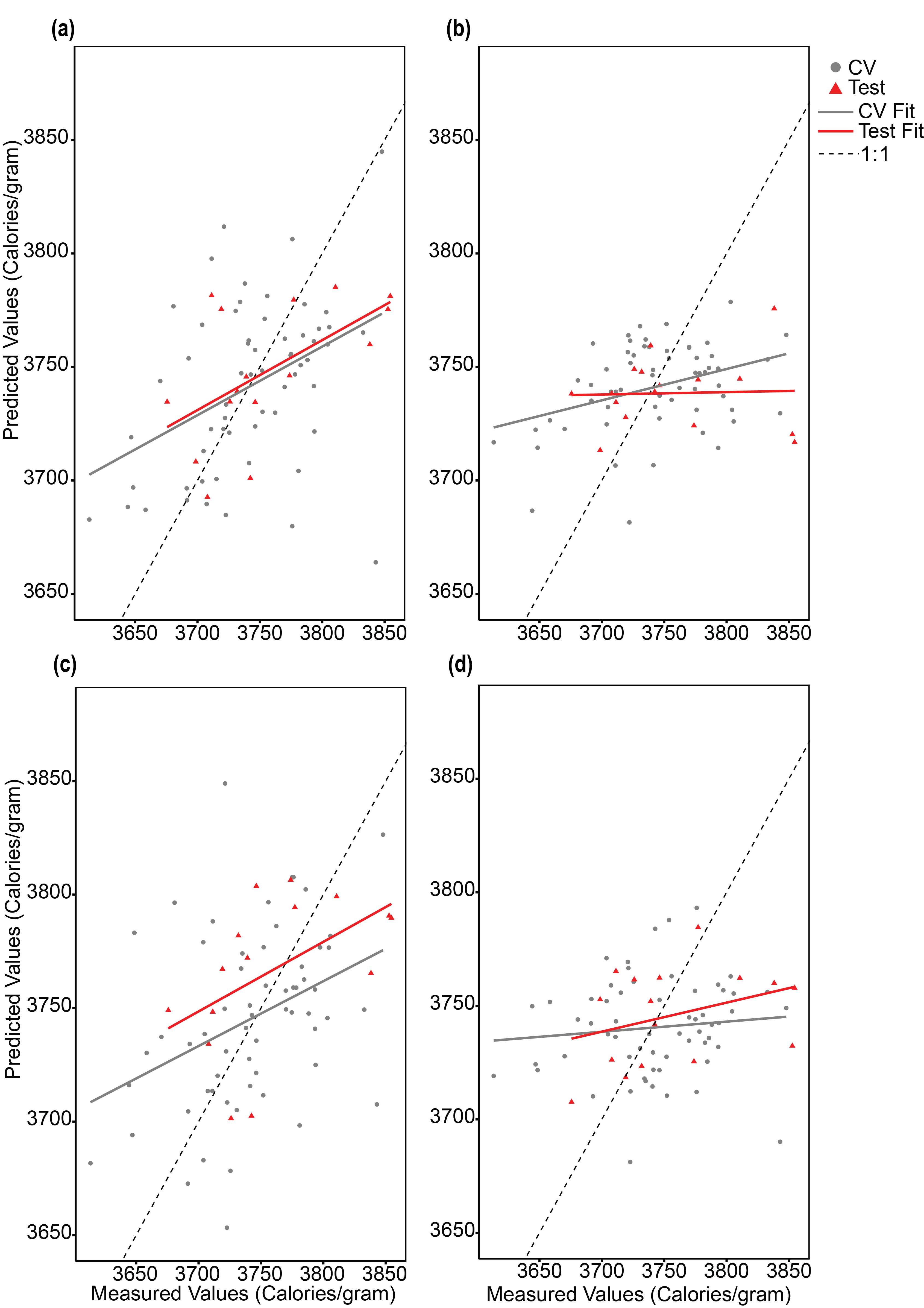

Supplement: Supplementary Figure 8 — Scatterplot of the lab-measured value vs. the image-predicted value of cal/g based on the full spectrum for half section results with at least n = 75. The data were split into a training and a test set consisting of 60 and 16 observations (75/25 split), respectively. The cross validation set is denoted by dot and test data set by triangle. Grey line represents the regression line for cross validation set, red line represents the test fit regression line, and the dotted line represents 1:1 regression fit. (A) Partial Least Squares Regression (PLSR) for fresh imaging data (B) Random Forest (RF) for fresh imaging data (C) Partial Least Squares Regression (PLSR) for oven dried imaging data (D) Random Forest (RF) for oven dried imaging data. The error metrics are available in Supplementary Table S3 . [file Image8.jpeg]
